# The myth of the Bayesian brain

**DOI:** 10.1007/s00421-025-05855-6

**Published:** 2025-06-26

**Authors:** Madhur Mangalam

**Affiliations:** Division of Biomechanics and Research Development, Department of Biomechanics, Center for Research in Human Movement Variability, Omaha, NE 68182 USA

**Keywords:** Bayesian brain hypothesis, Predictive coding, Free energy principle, Cognitive neuroscience, Theoretical neuroscience, Embodied cognition, Philosophy of neuroscience, Dynamic systems theory, Unfalsifiability

## Abstract

The Bayesian brain hypothesis—the idea that neural systems implement or approximate Bayesian inference—has become a dominant framework in cognitive neuroscience over the past two decades. While mathematically elegant and conceptually unifying, this paper argues that the hypothesis occupies an ambiguous territory between useful metaphor and testable, biologically plausible mechanistic explanation. We critically examine the key claims of the Bayesian brain hypothesis, highlighting issues of unfalsifiability, biological implausibility, and inconsistent empirical support. The framework’s remarkable flexibility in accommodating diverse findings raises concerns about its explanatory power, as models can often be adjusted post hoc to fit virtually any data pattern. We contrast the Bayesian approach with alternative frameworks, including dynamic systems theory, ecological psychology, and embodied cognition, which conceptualize prediction and adaptive behavior without recourse to probabilistic inference. Despite its limitations, the Bayesian brain hypothesis persists—driven less by empirical grounding than by its mathematical elegance, metaphorical power, and institutional momentum.

## The Bayesian boom

Every scientific revolution begins with elegant equations promising certainty, continues with increasingly baroque modifications to account for anomalies, and ends with a crowded citation list defending mathematical fictions that never quite mapped to biological reality.

The Bayesian brain hypothesis represents one of the most influential frameworks in contemporary cognitive science and neuroscience, having emerged as a dominant paradigm that has fundamentally reshaped how we conceptualize neural function (Doya 2007; Friston 2010; Knill and Pouget 2004). At its core lies a deceptively simple yet profound premise: the brain functions primarily as a sophisticated prediction machine that uses Bayesian inference to interpret sensory information and continuously update internal models of the world (Friston 2005; Lee and Mumford 2003; Rao and Ballard 1999). According to this perspective, neural processing is fundamentally concerned with minimizing prediction errors through probabilistic inference—essentially implementing something akin to Bayes’ theorem at various levels of neural organization, from individual cells to large-scale networks (Bogacz 2017; Friston 2010; Ma et al. 2006). This view constitutes a radical departure from traditional stimulus–response models by positioning the brain as an active, hypothesis-testing organ rather than a passive receiver of sensory information (Clark 2015; Friston 2010; Gregory 1980). This paradigm shift has sparked widespread enthusiasm and growing controversy, raising questions about whether the Bayesian brain is a genuine mechanistic account or merely a compelling metaphor (Bowers and Davis 2012; Colombo and Seriès 2012; Jones and Love 2011).

The intellectual lineage of this approach can be traced back to Helmholtz’s notion of perception as unconscious inference, but its modern formulation and meteoric rise to prominence occurred primarily during the early 2000 s. Building on earlier computational approaches to cognition, researchers like Karl Friston, Geoffrey Hinton, and Andy Clark have developed increasingly sophisticated mathematical models that describe perception, action, and learning in Bayesian terms. Friston’s Free Energy Principle (FEP) and its corollary, active inference, represents perhaps the most ambitious extension of this approach, aiming to derive principles of neural organization from thermodynamics and information theory. Meanwhile, Clark’s influential “whatever next” paper positioned predictive processing as a potential “grand unified theory of the brain,” while Hinton’s work on generative models established crucial connections between Bayesian frameworks and neural network implementations. Together, these researchers and many others have transformed what began as a specialized approach in vision research into an expansive paradigm that now encompasses virtually all domains of cognition.

The explanatory scope of the Bayesian brain hypothesis has expanded at a remarkable pace, extending beyond perception to encompass action, attention, learning, decision-making, and even psychiatric disorders. What makes this framework particularly appealing is its mathematical elegance and apparent unifying power—it promises to provide a common language and set of computational principles that bridge traditionally separate domains of neurobiology, psychology, and psychiatry under a single coherent framework. For example, conditions like schizophrenia have been reinterpreted as disorders of precision-weighting in hierarchical predictive coding networks, while phenomena like the placebo effect have been reconceptualized as manifestations of strong perceptual priors influencing bodily states. This expansive reach has elevated the Bayesian brain from merely another theory of neural function to a paradigm-defining framework that shapes research questions, experimental designs, and interpretations across multiple fields. Yet, this very expansiveness—its capacity to explain nearly everything—has led some to question whether the Bayesian brain risks becoming a theory of everything that, paradoxically, explains nothing in a scientifically constraining way.

These concerns are not unprecedented. A growing body of critical scholarship has questioned the empirical, mechanistic, and conceptual foundations of Bayesian frameworks in cognitive science and neuroscience. For example, Bowers and Davis (2012) warned that many Bayesian models are too flexible to be falsifiable and often rely on post hoc parameter fitting rather than making risky predictions. Jones and Love (2011) argued that rational Bayesian models are typically underconstrained, often neglect mechanistic specificity, and risk functioning as metaphors rather than scientific theories. Colombo and Seriès (2012) further noted that many such models serve primarily as descriptive tools and lack correspondence with identifiable neural mechanisms, making the inference from Bayesian behavior to Bayesian brains highly tenuous. Other critiques have highlighted the difficulty of empirically constraining priors and loss functions in perceptual models (Colombo 2018), and the problematic metaphysical overreach of the Free Energy Principle in its attempts to explain life, cognition, and action through a single variational formalism (Colombo and Wright 2021). Most directly aligned with our critique, Williams (2018); Williams and Colling (2018); Williams (2020) argued that the FEP’s universal scope and mathematical generality render it effectively unfalsifiable. His work has specifically shown that FEP avoids empirical refutation by shifting interpretive ground across levels of analysis. Our work builds on these critiques by systematically analyzing how rhetorical strategies, metaphor–mechanism slippage, and institutional incentives have jointly reinforced the persistence of an unfalsifiable and biologically implausible framework.

The mathematical sophistication of Bayesian approaches has lent them considerable prestige within the increasingly computational landscape of neuroscience. Complex behaviors and neural responses can be formalized in terms of probability distributions, likelihood functions, and prior beliefs—bringing a satisfying rigor to domains previously characterized by descriptive or phenomenological accounts. This quantitative precision allows researchers to derive specific, testable predictions and to fit complex models to behavioral and neural data. The approach’s success in modeling certain perceptual phenomena—such as visual illusions, multisensory integration, and context effects—has bolstered confidence in its broader applicability, leading to what might fairly be called a “Bayesian boom” in neuroscientific research. However, this surge in popularity also raises important questions about whether the mathematical elegance of Bayesian models is being mistaken for empirical adequacy or mechanistic insight.

Despite its undeniable popularity and apparent explanatory power, this paper argues that the Bayesian brain hypothesis occupies an ambiguous conceptual territory between useful metaphor and legitimate mechanistic explanation. While it has undoubtedly generated productive research programs and compelling interpretations of neural function, its status as an explanatory theory remains deeply problematic when subjected to critical scrutiny. The challenge is not merely technical but conceptual—concerning the very nature of scientific explanation in neuroscience and what constitutes a satisfactory account of neural function. The following sections will demonstrate that when pressed on its mechanistic claims, proponents of the Bayesian brain often retreat to metaphor or abstraction; when challenged on its metaphorical looseness, they reassert its mechanistic aspirations and point to implementation details. This conceptual slippage, I argue, threatens the scientific integrity of the approach and requires critical examination to assess whether it genuinely explains brain function or merely sustains the appearance of explanation—whether it remains a viable model or has already crossed the line into scientific myth.

The Bayesian perspective has also transformed how we understand the relationship between brain, body, and environment (Allen and Friston 2018; Clark 2015; Kirchhoff and Kiverstein 2019). By framing perception as inference and action as a means of testing perceptual hypotheses, it challenges traditional boundaries between sensing and doing (Clark 2013; Hohwy 2013; Seth 2014). This has profound implications for how we conceptualize embodied cognition and the extended mind—suggesting that the brain’s primary purpose is not to represent the world accurately but to facilitate successful interaction with it through prediction (Clark 2015; Gallagher and Allen 2018; Kirchhoff 2018). Yet this reformulation, however compelling, raises profound questions about implementation: how could neural tissue actually encode probability distributions, perform Bayesian updates, or represent precision estimates (Fiser et al. 2010; Pouget et al. 2013; Sanborn and Chater 2016)? These questions of biological plausibility stand in tension with the framework’s mathematical elegance, creating a gap between theoretical aspiration and mechanistic reality that remains inadequately addressed in much of the literature (Bowers and Davis 2012; Nelson et al. 2018; Williams and Colling 2018).

Moreover, the social dynamics of scientific research have contributed to the framework’s rapid ascendancy (Kuhn 1962; Latour 1987; Smaldino and McElreath 2016). The Bayesian approach offers researchers powerful mathematical formalisms, computational models, and a common vocabulary that facilitates collaboration across disciplines (Clark 2013; Griffiths et al. 2010; Hohwy 2013). Career incentives reward those working within influential paradigms, and funding agencies often prioritize research that builds on established frameworks rather than those that challenge them fundamentally (Nelson et al. 2018; Romero 2017; Smaldino and McElreath 2016). These sociological factors, while external to the scientific content of the theory itself, nevertheless, shape how the Bayesian brain hypothesis has been developed, evaluated, and institutionalized within neuroscience (Kuhn 1962; Latour 1987; Longino 2020). Understanding these dynamics is crucial for assessing not just the theory’s truth but its persistence and influence despite significant conceptual and empirical challenges (Ioannidis 2005; Smaldino and McElreath 2016).

These considerations raise deep concerns about the explanatory coherence and empirical viability of the Bayesian brain hypothesis. Despite its widespread adoption and mathematical sophistication, the framework often oscillates between metaphor and mechanism, adapting flexibly to accommodate contradictory findings without clear constraints. Its ability to unify diverse domains has become both its greatest strength and its most serious vulnerability. What appears at first as theoretical elegance may, on closer inspection, reflect a troubling looseness of formulation—one that undermines the framework’s explanatory coherence and opens the door to uncritical adoption. To assess its actual scientific value, we must rigorously investigate its conceptual commitments, empirical support, and biological plausibility.

While this paper aims to provide a rigorous philosophical and scientific critique of the Bayesian brain hypothesis and the FEP, we acknowledge that some of the language may strike readers as unusually direct. This is intentional. The rhetorical tone—at times pointed or sharply critical—is meant to reflect the seriousness of the epistemological and institutional issues at stake. Specifically, we aim to highlight how certain theoretical frameworks can achieve disproportionate influence despite persistent conceptual and empirical shortcomings. This style follows a long tradition in philosophy of science, where direct critique is often necessary to expose unfalsifiable or overextended explanatory systems that resist correction through standard empirical means. Rather than targeting individuals or communities, the tone is directed at systemic tendencies in contemporary neuroscience to conflate metaphor with mechanism, and mathematical elegance with biological plausibility. We have nonetheless taken care to revise language where it may have been overly dismissive or needlessly inflammatory, and we hope readers will interpret the critique as a principled intervention, not a polemic.

The paper proceeds as follows to clarify the stakes of this critique and provide readers with a clear guide through the argument. First, we unpack the core theoretical claims of the Bayesian brain hypothesis and its associated frameworks, including predictive coding and the Free Energy Principle. Next, we examine the ambiguity between metaphor and mechanism that characterizes many Bayesian models, followed by an in-depth analysis of their unfalsifiability and over-flexibility. We then turn to the biological and neurophysiological limitations of the hypothesis, including problems of computational and metabolic feasibility, implementation challenges, and mismatches with empirical data. After outlining the non-Gaussian statistical properties of neural dynamics as a fundamental challenge to Bayesian assumptions, we introduce alternative frameworks—dynamic systems theory, ecological psychology, and embodied cognition—that offer more biologically plausible accounts of perception and action. The manuscript concludes with a fictional dialogue that crystallizes the core conceptual tensions and reflects on why the Bayesian metaphor persists despite its empirical and mechanistic shortcomings. We thus draw a clear boundary between scientific explanation and seductive abstraction.

## What is the Bayesian brain claiming?

The danger of a good idea is not that it fails to explain anything, but that it can be twisted to explain everything. When a theory grows flexible enough to encompass candle flames and cortical columns under the same mathematical umbrella, perhaps we should question not its scope but its substance.

Before we can fully assess these concerns, we must first clarify what the Bayesian brain hypothesis actually claims. While variations exist across different research programs, the core commitments generally include:

### Perception as probabilistic inference

**Fig. 1:**
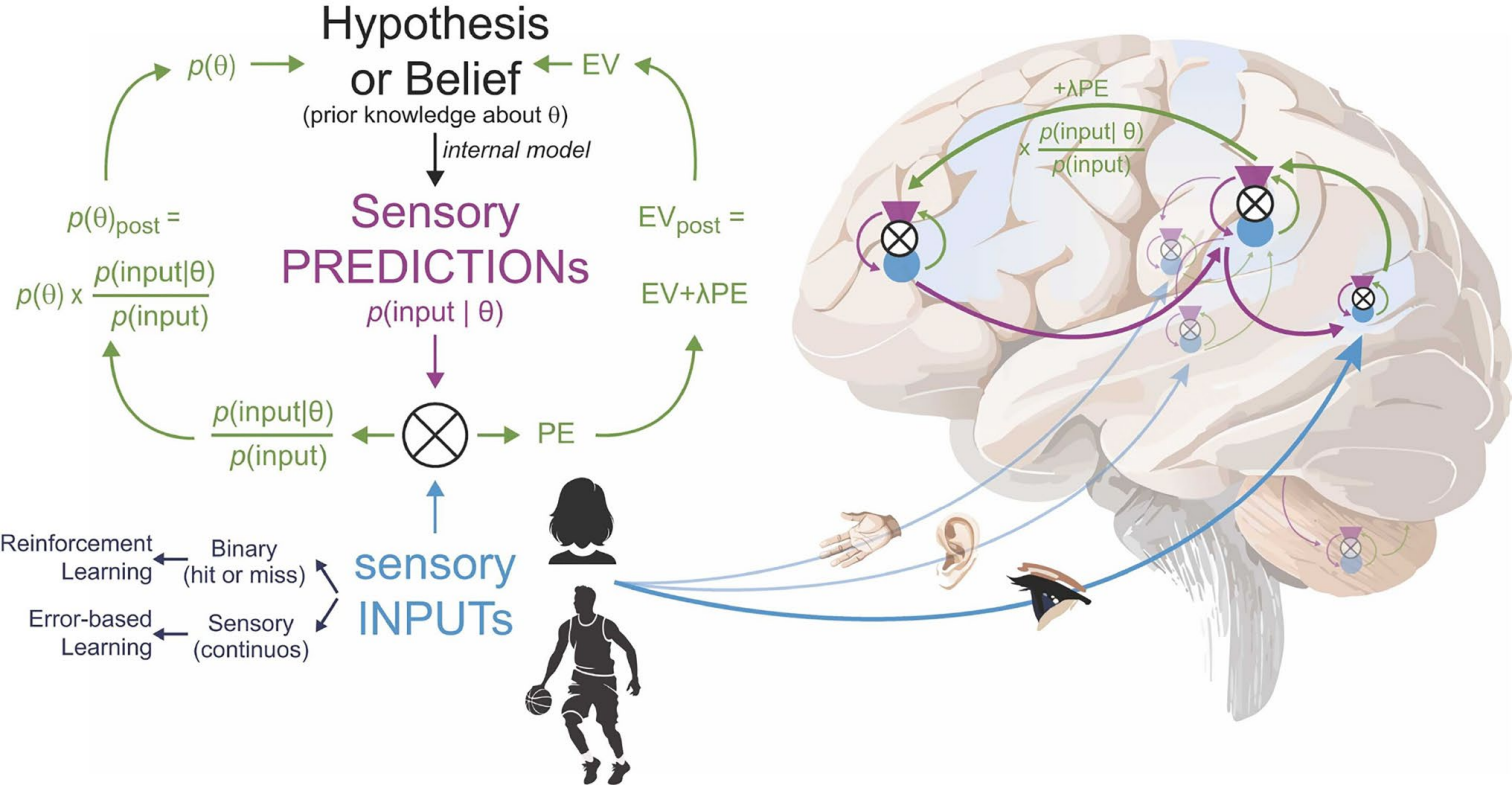
A schematic representation of the Bayesian brain hypothesis, illustrating how the brain integrates prior beliefs (hypotheses) with incoming sensory inputs to generate predictions and update internal models. Sensory inputs (blue arrows) from the environment—including visual, auditory, and proprioceptive signals—are compared against internally generated sensory predictions (purple arrows) derived from prior beliefs about the world. Prediction errors (PE), calculated as the discrepancy between expected and actual input, are used to update beliefs (green arrows) and expected value (EV), adjusting the brain’s internal model through processes such as error-based learning and reinforcement learning. The brain minimizes prediction error, refining its model to interpret better and anticipate sensory information. This framework is foundational to predictive coding theories and active inference in neuroscience. Figure reproduced from Keysers et al. (2024). Creative Commons Attribution 4.0 International (CC BY 4.0)

The Bayesian brain frames perception not as a passive reception of sensory data, but as an active process of probabilistic inference (Knill and Pouget 2004; Friston 2005; Rao and Ballard 1999). Sensory inputs are viewed as evidence that the brain uses to test and update its internal models of the world. What we perceive is not the world directly, but the brain’s best prediction about the causes of its sensory inputs, constrained by prior expectations and the likelihood of specific sensory data given different possible causes (this process is often illustrated schematically in models of predictive coding, where prior beliefs are combined with incoming sensory input to generate perceptual estimates and error signals, as shown in **Fig**. 1).

### Action as prediction-error minimization

Under this framework, motor actions are seen as attempts to minimize prediction errors by altering sensory inputs to align with predictions, rather than adjusting predictions to match inputs as in perception (Adams et al. 2013; Brown et al. 2013; Friston 2011). This perspective reframes motor control as a process analogous to perception, with both understood as forms of Bayesian inference. The key distinction lies in the direction of inference: perception updates predictions based on inputs, while action modifies inputs to fulfill predictions.

### Learning as Bayesian belief updating

In the Bayesian framework, learning is the ongoing process of updating internal models in response to mismatches between predicted and actual sensory input—that is, prediction errors. This updating is presumed to follow Bayes’ rule, which combines prior beliefs with likelihood functions to generate posterior beliefs (Behrens et al. 2007; Mathys et al. 2011; Tenenbaum et al. 2006). Over time, this inferential mechanism is thought to refine the brain’s generative models, enabling progressively more accurate predictions about the structure and dynamics of the external world.

These core claims have been elaborated and extended in several related frameworks, most notably:

**Predictive coding** models describe how prediction errors might be computed and propagated through hierarchical neural systems, with higher levels providing predictions to lower levels and receiving error signals in return (Bastos et al. 2012; Clark 2013; Rao and Ballard 1999).

**The Free Energy Principle (FEP)** attempts to unify perception, action, and learning under a single imperative: minimizing variational free energy, which is claimed to approximate prediction error or surprise over time (Friston et al. 2006; Friston 2010; Parr and Friston 2019).

**Active inference** extends these ideas to account for decision-making and planning, suggesting that agents select actions that minimize expected free energy or maximize expected information gain (Friston et al. 2011, 2017; Parr et al. 2020).

While these frameworks vary in mathematical details and scope, they share the fundamental assumption that Bayesian inference, or something functionally equivalent, constitutes the basic operating principle of neural systems. To clarify the rhetorical and conceptual slippage across different implementations of the Bayesian brain hypothesis, **Table** 1 summarizes core terms and how their meanings vary by context, model level, and narrative intent. **Table** 2 extends this point by showing how these same terms often shift meaning entirely depending on context—serving simultaneously technical, rhetorical, and metaphorical functions.

**Table 1:** Glossary of core terms in the Bayesian brain literature. These terms are widely used across predictive coding and active inference models, often with flexible or contradictory meanings depending on the context. Readers are encouraged to examine how each term is operationalized—or left undefined—in specific studies

Term	Stated meaning	Operational usage	Convenient flexibility
**Prior**	Belief about a hidden cause or state	Often fitted post hoc from data; rarely observable directly	Can be adjusted arbitrarily to explain behavioral variance
**Likelihood**	Probability of data given a state	Used in model-fitting to simulate expected input	Rarely derived from actual sensory noise or measurement properties
**Posterior**	Updated belief after observing data	Equated with perceptual content or motor intent	Sometimes collapses back into the prior depending on results
**Prediction error**	Difference between predicted and observed input	Inferred from fMRI or EEG correlates	Broad enough to explain nearly any neural response
**Precision**	Confidence (inverse variance) on a prediction error	Said to modulate neural gain or synaptic weights	Alternately interpreted as attention, salience, or relevance
**Generative model**	Internal model that simulates sensory input	Invoked to explain perception, action, and imagination	Often remains a black box between equations and neurons
**Free energy**	Bound on surprise or model evidence	Optimization target for Bayesian agents	Used metaphorically, mathematically, and metaphysically
**Active inference**	Acting to minimize expected free energy	Justifies perception, action, planning, and emotion	May explain everything while predicting nothing
**Surprise**	Improbability of an observation	Linked to learning, novelty, or model update	Sometimes psychological, sometimes mathematical, always fuzzy
**Markov blanket**	Statistical boundary between system and environment	Applied to neurons, organs, and even candle flames	Becomes metaphysical when stretched too far

**Table 2:** Polysemy as a feature: How core terms shift shape across contexts. Terms commonly used in Bayesian frameworks often take on different meanings depending on rhetorical or theoretical need

Term	Context A	Context B
**Precision**	Neural gain modulation (e.g., V1 BOLD signal)	Subjective confidence or attentional weighting
**Free energy**	Surprise minimization (in theory)	Surprise rebranding (in practice)
**Surprise**	Kullback–Leibler (KL) divergence between model and input	Phenomenological “Whoa!” moments in psychology
**Priors**	Built-in generative model constraints	Flexible narrative device to explain deviant data
**Prediction error**	Signal mismatch at sensory cortex	Any unexpected effect in behavior or physiology

Notably, “Bayesian brain” theories vary widely in their claims, from literal implementations of Bayes’ rule at the neural circuit level (strong mechanistic models), to computational-level descriptions that treat Bayesian inference more as an “as-if” convenience. Some researchers adopt approximate sampling approaches (e.g., Monte Carlo sampling in neural populations), others discuss Bayesian integration only at the behavioral level, and still others envision a purely metaphorical role. This heterogeneity makes it crucial to specify which “Bayesian flavor” is under discussion.

While the core claims of the Bayesian brain hypothesis are relatively clear, their status as actual mechanistic explanations remains deeply contested and persistently unresolved. This ambiguity reflects a broader and longstanding issue in theoretical neuroscience: the potential conflation of metaphorical descriptions with genuine, biologically grounded accounts of neural function. To address this issue meaningfully, we must carefully and explicitly distinguish between these different modes of explanation—not only in language, but in empirical commitments and evidentiary standards.

## From metaphor to mechanism—or myth

A metaphor is a promise. A mechanism is its fulfillment. A myth is what remains when the promise is never kept.

The Bayesian brain hypothesis did not begin as a mechanism. Like many influential scientific ideas, it began as a metaphor—a conceptual lens through which to reinterpret core cognitive processes such as perception, action, and learning. Rather than presenting a fully articulated model of neural function grounded in biological detail, the hypothesis offered a broad, unifying framework that cast the brain as an inference engine, continuously generating and updating probabilistic models of the world. This framing provided a flexible alternative to classical stimulus–response models, allowing for the reimagination of brain function as prediction, uncertainty, and statistical optimization. As a heuristic, the idea of the brain as a “Bayesian inference engine” proved powerful: it offered a narrative capable of organizing diverse phenomena across levels of analysis. Yet metaphors in science carry an implicit contract—they must eventually be translated into mechanistic explanations that are biologically plausible and empirically testable (Craver 2007). When they are not—when they continue to shape theory and research despite lacking clear implementation or falsifiability—they cross a threshold and become self-sealing narratives: rhetorically compelling, yet empirically ungrounded.

To make this distinction precise, we briefly define three conceptual categories:

1. **Metaphor:** A metaphor is an analogical device that maps one domain of understanding onto another (Lakoff and Johnson 1980). In science, metaphors are often indispensable for theory development, serving as scaffolding for the construction of models or hypotheses (Hesse 1970). Yet a metaphor is not an explanation: it is a guide, not a ground.
2. **Mechanism:** A mechanism, by contrast, is a causally specific, empirically tractable process embedded in physical systems. A mechanistic explanation identifies components, operations, and their organization in producing a phenomenon. It is the standard by which claims about brain function must ultimately be judged (Bechtel 2011; Craver 2007; Machamer et al. 2000).
3. **Myth:** A myth is what remains when a metaphor persists without becoming a mechanism. It is a narrative that resists empirical challenge, flexibly absorbs contradictory findings, and sustains belief through rhetorical appeal rather than evidential support. Myths in science are not necessarily false—but they are unfalsifiable, and thus epistemically inert.

This taxonomy clarifies what is at stake in the ongoing debate over the Bayesian brain hypothesis by distinguishing between metaphor, mechanism, and myth. The predictive coding framework, and later the Free Energy Principle, did not begin as mechanistic models but as evocative metaphors—mathematical formalisms that invited reinterpretation of neural function in probabilistic terms (Clark 2013; Knill and Pouget 2004; Friston et al. 2006; Friston 2010; Hohwy 2013). These metaphors proved highly generative: they inspired theoretical innovation, computational simulations, and reinterpretations of empirical findings across diverse cognitive domains. Yet generativity alone is not sufficient for scientific legitimacy. In many cases, these constructs have failed to evolve into biologically grounded mechanisms. The internal generative model, precision-weighted prediction error, and the Markov blanket remain metaphorical placeholders unless and until they are tied to specific, testable neural substrates and causal processes—as some approximation-based models attempt, though often without sufficient constraint or empirical validation. Until then, they function less as mechanistic explanations and more as durable narrative devices—mathematically fluent, rhetorically compelling, and empirically ambiguous.

Moreover, when these metaphors are challenged, proponents often retreat to abstraction rather than mechanism. When neural data fails to support prediction error coding, the theory shifts to another level of analysis. When behavior contradicts Bayesian optimality, the priors are redefined. This flexibility is not a sign of robustness; it is a sign of rhetorical insulation. As Popper (2014) warned, a theory that cannot be falsified—because it can explain any outcome—ceases to be scientific.

The problem is not that the Bayesian brain began as a metaphor; all scientific theories do. The problem is that it has remained one—while being defended as if it were a mechanism. It is this unresolved status that turns the metaphor into a myth.

In the following sections, we explore how this process of mythologization unfolds across several key domains of cognitive neuroscience, including perception, motor control, psychiatric modeling, and artificial intelligence. Each of these domains has been reshaped by the Bayesian framework’s rhetorical and mathematical appeal, yet often without corresponding empirical or mechanistic specificity. We argue that unless proponents can rigorously demonstrate how their constructs map onto identifiable neural mechanisms, the Bayesian brain hypothesis must be reclassified not as a genuine explanation of brain function, but as a durable, aesthetically appealing, and ultimately empirically hollow metaphor.

## The “as-if” problem

To say the brain behaves “as if” it were a Bayesian inference engine is not an explanation—it is an abdication. It is the polite fiction we tell ourselves when the equations look good but the biology refuses to cooperate. It is a way to sound mechanistic while saying nothing mechanistic at all.

The Bayesian brain hypothesis frequently operates in an “as-if” mode of explanation: the brain acts *as if* it were performing Bayesian inference, without claiming that actual Bayesian computations are implemented in neural tissue (Colombo and Seriès 2012; Danks 2014). This linguistic framing is not merely stylistic but reveals a fundamental ambiguity in the theory’s explanatory status (cf. Ramsey 2007). It allows theorists to borrow the authority of mathematics while avoiding commitment to the messiness of biological implementation.

The “as-if” problem manifests in several ways. First, descriptions of neural processes invariably slip into intentional language—neurons “expect,” “predict,” or “represent” probability distributions—suggesting cognitive operations that the theory simultaneously disavows at the implementation level (Brette 2019). Second, the very mathematics of Bayesian inference requires computation over explicit probability distributions, yet proponents often claim that the brain need not explicitly represent these distributions (Sprevak and Smith 2023). This leads to an unstable oscillation between literal and metaphorical interpretations depending on the empirical context. What begins as a theoretical convenience quickly becomes a rhetorical strategy.

Most problematically, the “as-if” framing allows the theory to preserve its narrative appeal and scientific prestige while systematically evading clear mechanistic commitments, except in a limited number of computational neuroscience implementations that warrant separate evaluation. When neural data fails to align with Bayesian predictions, the retreat to “as-if” terminology preserves the framework’s rhetorical force despite remaining detached from biological reality (Bowers and Davis 2012; Jones and Love 2011). This linguistic slippage becomes particularly acute in expressions like “the brain minimizes prediction error” or “neurons encode precision weights”—phrases implying mechanistic claims while remaining shielded from falsification by their metaphorical status (Litwin and Miłkowski 2020).

The “as-if” problem thus reveals a deeper issue: without a clear commitment to implementational specificity, Bayesian brain models risk becoming what Dennett (1987) calls “free-floating rationales”—explanations that identify computational functions without constraining how those functions are realized in physical systems. To move beyond this impasse requires abandoning the comfort of “as-if” terminology and specifying exactly what neural operations would constitute—not merely simulate or approximate—Bayesian inference (Zednik and Jäkel 2016).

This reliance on “as-if” reasoning thus functions not merely as a linguistic shortcut but as a conceptual buffer—protecting the framework from empirical scrutiny while preserving its rhetorical coherence. But what begins as a metaphorical convenience gradually metastasizes into explanatory ambiguity. As we will see in the next section, this ambiguity is not an isolated issue but symptomatic of a deeper theoretical tendency: the conflation of metaphor with mechanism, which threatens to transform a once-generative idea into a self-insulating scientific myth.

## Metaphor, mechanism, or myth: The conceptual drift of the Bayesian brain

A metaphor becomes a myth the moment we stop interrogating its limitations and start defending its universality. The Bayesian brain was born as an analogy, matured into a paradigm, and calcified into dogma—all without ever demonstrating its biological plausibility.

This section draws on Marr’s well-known framework, distinguishing computational, algorithmic, and implementational levels of explanation (Marr 1982). We do not argue against the utility of Bayesian models at the computational level, where they often serve as useful abstractions for describing task structure or statistical inference. Rather, our critique focuses on the conceptual slippage between levels: metaphorical or computational claims are often implicitly treated as if they entailed mechanistic commitments, without sufficient constraint at the algorithmic or implementational level. It is this rhetorical and explanatory ambiguity—not probabilistic modeling per se—that we identify as the central problem. Clarifying this distinction is essential for evaluating the scientific status of the framework.

Whether due to deliberate flexibility or a lack of conceptual clarity, the explanatory status of the Bayesian brain hypothesis remains surprisingly ambiguous—even as it is often presented with the mathematical formalism of a fully developed scientific theory. Its apparent precision can sometimes mask a lack of mechanistic clarity, allowing the framework to shift fluidly between levels of description without clear empirical constraint. When closely examined, many “Bayesian brain” models function not as process models—particularly in cognitive neuroscience and psychiatry—describing actual neural mechanisms, but as statistical descriptions that characterize behavioral or neural data in Bayesian terms, often retrospectively (Bowers and Davis 2012; Jones and Love 2011).

As noted above, Bayesian accounts span a wide spectrum of interpretations, ranging from purely metaphorical frameworks that offer broad conceptual guidance to computational-level approximations that describe the functional goals of perception and cognition and, finally, to strong mechanistic claims that purport to specify the actual neural implementations of inference in the brain. This ambiguity is particularly evident in the interpretation of neural activity. Consider studies that identify neural signals as representing “prediction errors” or “prior expectations.” These interpretations frequently rest on correlational evidence (O’Reilly et al. 2012; Poldrack 2011). The inference from correlation to representation is not always substantiated, yet it is often rhetorically framed as evidence of implementation. If neural activity correlates with values derived from a Bayesian model fitted to behavioral data, it is claimed that this region “represents” or “encodes” the corresponding Bayesian construct. This reflects a broader issue of reverse inference: the fact that a Bayesian model fits observed data does not imply that the brain necessarily implements Bayes’ rule or explicitly represents Bayesian quantities.

A central concern here is reification—treating mathematical constructs as physical entities or neurobiological processes (Krakauer et al. 2017). Priors, likelihoods, and posteriors are mathematical terms that help us describe probabilistic relationships, but it is unclear whether the brain carves itself at these particular joints or manipulates such quantities in any explicit form. Neural activity that correlates with prediction errors might emerge from underlying dynamics or structural constraints that may not correspond directly to explicit Bayesian calculations.

When challenged on these points, proponents of the Bayesian brain often pivot between two positions. On one hand, they may retreat to a weaker claim: Bayesian models provide a useful “as if” description, without committing to specific implementation details. On the other hand, they may assert stronger mechanistic claims when presenting evidence that seems to align with Bayesian predictions. This conceptual ambiguity—between abstraction and implementation, metaphor and mechanism—allows the framework to absorb contradictory findings without penalty, insulating it from empirical falsification while maintaining explanatory appeal. This conceptual flexibility makes it difficult to pin down exactly what the hypothesis is claiming about actual brain function.

The metaphor-mechanism distinction matters deeply. If the Bayesian brain is primarily a metaphor or high-level description, it should not be evaluated on its mechanistic accuracy but on its heuristic value in generating testable predictions. However, if it makes mechanistic claims, it must specify how abstract Bayesian computations map onto biological processes and neural architectures. Appeals to Marr ’s (1982) computational level does not absolve the framework from empirical accountability. If the computational-level theory is too flexible to be falsified, or if it lacks algorithmic and implementational constraints, then its scientific utility diminishes. A coherent theory must connect its computational goals to biologically plausible processes and allow for empirical disconfirmation at each level. Without this clarity, the hypothesis risks becoming what philosopher Karl Popper would call a “metaphysical research program” (Popper 2014)—influential but ultimately unfalsifiable.

### Falsifiability criteria for the Bayesian brain hypothesis

To evaluate the Bayesian brain hypothesis as a scientific theory, it must generate predictions that are specific enough to be disproven by empirical evidence. The following conditions would, in principle, falsify core claims of the hypothesis or its associated frameworks (e.g., predictive coding, active inference):

1. **Absence of prediction error signatures:** Neural populations hypothesized to encode prediction errors (e.g., mismatch responses in sensory cortices) should systematically differ under conditions of violated expectations (Egner and Summerfield 2013). If no such differences are observed—even when priors are explicitly manipulated—this challenges the predictive coding framework.
2. **Failure to detect prior-driven modulation:** The theory predicts that prior expectations modulate neural activity and behavioral outcomes. Experiments designed to isolate prior influence (e.g., cue-based probabilistic expectations) that show no such modulation undermine Bayesian assumptions (Teufel and Fletcher 2020).
3. **Non-hierarchical neural responses:** Predictive coding models assume hierarchical processing. If top-down predictions and bottom-up errors do not interact as predicted—e.g., if perturbations to higher-order areas do not affect lower-level error responses—this violates core architectural assumptions (Clark 2013).
4. **Lack of precision weighting evidence:** A key claim is that the brain dynamically adjusts the weight of prediction errors based on uncertainty. Empirical evidence that uncertainty manipulation does not modulate neural gain or connectivity (e.g., through fMRI or electrophysiological signatures) would contradict this mechanism.
5. **Violation of behavioral Bayesian optimality:** In tightly controlled tasks where prior probabilities and likelihoods are explicitly defined, persistent deviations from Bayesian-optimal behavior—unexplainable by noise, heuristics, or approximation—would falsify normative claims (Rahnev and Denison 2018).
6. **Disconfirmation of neural implementations:** Proposed neural implementations (e.g., probabilistic population coding, sampling-based approximations) must be empirically verified. If studies fail to identify populations or dynamics consistent with these computational roles, the mechanistic plausibility of the framework is weakened.
7. **Predictive failure in clinical models:** If Bayesian-based explanations of psychiatric disorders (e.g., schizophrenia as altered precision weighting) fail to predict symptom patterns, treatment outcomes, or neural correlates in out-of-sample datasets, the theory’s applied value diminishes (Fletcher and Frith 2009).

Together, these criteria illustrate that Bayesian brain models must be held to the same standards of empirical testability as any scientific theory. Absent clear predictions and falsifiability conditions, the framework risks functioning as a flexible narrative rather than a mechanistic explanation. Table 3 illustrates how common responses to disconfirming data—though rhetorically effective—gradually dissolve the framework’s empirical bite.

**Table 3:** Adapt or die? How flexibility undermines falsifiability (Danks 2014). A sample of common responses to disconfirming data and why each reduces the empirical bite of the theory. *Note:* These criteria apply most strongly to generalized or flexible Bayesian frameworks; some tightly constrained Bayesian models may offer stronger empirical grounding

Response to contradiction	How it preserves the theory	Why it undermines science
Adjust priors to fit data	Retains Bayesian framing	Priors become unfalsifiable parameters
Invoke suboptimal inference	Absorbs errors into theory	Prevents theory from being proven wrong
Retreat to “as-if” model	Evades mechanistic commitment	Confuses metaphor for mechanism
Shift levels of analysis	Reframes failure at one level as success at another	Eliminates clear prediction accountability

These criteria are not intended to specify detailed experimental paradigms, but rather to highlight the theoretical commitments that must be clarified for the Bayesian brain hypothesis to function as a testable scientific theory. They serve to delineate the boundaries of falsifiability, without presupposing that such boundaries have yet been meaningfully enforced in current empirical practice. Clarifying these boundaries is a necessary step before meaningful experimental testing can even begin.

**Concrete examples.** The abstract concerns raised above are not merely theoretical; they are reflected in the empirical literature where predictive processing and FEP-based models are frequently invoked. For example, in the clinical domain, Bayesian models of schizophrenia posit that positive symptoms emerge from the altered precision weighting of prediction errors (Adams et al. 2013; Fletcher and Frith 2009; Friston et al. 2014). While conceptually appealing, such models are often underconstrained and rarely generate falsifiable predictions about symptom specificity or treatment outcomes. In perceptual neuroscience, auditory mismatch negativity (MMN) is frequently interpreted within a predictive coding framework (Garrido et al. 2009; Teufel et al. 2013; Teufel and Fletcher 2020). Yet, similar neural responses can be explained by simpler mechanisms such as adaptation or dynamic filtering, making the inference to hierarchical prediction error computation questionable. Each example concretely illustrates the theoretical concerns raised above. Finally, in cue integration paradigms, such as the classic visual–haptic study by Ernst and Banks (2002), retrospective model-fitting often justifies Bayesian optimality post hoc, as priors and likelihoods are typically inferred from the behavioral data they purport to explain, rather than derived independently. These cases illustrate how flexible interpretation and post hoc adjustment—hallmarks of conceptual slippage—play out in practical modeling scenarios.

**A note on descriptive utility.** While this paper strongly critiques the mechanistic and empirical claims of the Bayesian brain hypothesis, it is important to acknowledge that Bayesian models can still serve a useful descriptive function in certain contexts (Jones and Love 2011). Their ability to fit data and formalize uncertainty has made them valuable formalisms for summarizing behavioral trends or designing experimental paradigms. However, such uses should be clearly delineated from claims about neural implementation. A Bayesian model that captures behavior is not necessarily evidence for Bayesian computation in the brain. Conflating descriptive adequacy with mechanistic validity remains one of the central confusions this paper seeks to clarify.

## Unfalsifiability and flexibility

A theory that adapts to all outcomes predicts none. When priors can be adjusted post-hoc to accommodate any observation, and precision parameters can explain away any deviation, we are no longer doing science—we risk falling into an overly flexible interpretative framework.

Perhaps the most troubling aspect of the Bayesian brain hypothesis is its extraordinary flexibility in accommodating a wide range of empirical findings, including those that appear contradictory. By adjusting free parameters—such as priors, likelihood functions, noise distributions, or assumed processing levels—Bayesian models—particularly in their loosely specified, post hoc applications—an often be tailored to fit virtually any observed data pattern (Bowers and Davis 2012; Jones and Love 2011). This flexibility manifests in several ways:

First, when behavioral data fail to conform to the predictions of Bayesian optimality, researchers frequently appeal to auxiliary assumptions—such as “suboptimal” priors, cognitive capacity limitations, or the use of approximation algorithms—to account for these deviations (Rahnev and Denison 2018; Sanborn and Chater 2016). While such adjustments may be reasonable and necessary within a particular experimental contexts, their cumulative effect renders the framework remarkably elastic and resistant to empirical challenge. As a result, almost any observed behavior—no matter how inconsistent with normative expectations—can be retroactively interpreted as “Bayesian” in some qualified, metaphorical, or indirect sense, thereby blurring the boundary between explanation and rationalization.

Second, when neural data fail to align with predicted Bayesian patterns, proponents can readily shift the theoretical focus between different levels of analysis. Proponents might even argue that individual neurons are not performing Bayesian computations, but that such computations emerge at the level of neural populations. Alternatively, the Bayesian framework may be invoked at a more abstract, computational level, where its validity does not depend on direct neural implementation (Colombo and Seriès 2012; Kiefer and Hohwy 2018). Particularly telling is how predictive coding advocates responded when single-unit recordings in macaque IT cortex by Kaliukhovich and Vogels (2014) failed to show the expected repetition suppression effects. Rather than interpreting this as a challenge to the theory, they argued that Bayesian processes might operate at different spatial or temporal scales, or that such effects only emerge under specific task conditions (Walsh et al. 2020).

Third, the hypothesis spans multiple timescales, ranging from moment-to-moment perceptual processes to long-term evolutionary dynamics. This expansive scope allows proponents to shift the explanatory emphasis when challenged. For instance, if current neural activity does not appear explicitly Bayesian, it may be reinterpreted as approximating Bayesian solutions that emerged through evolutionary or developmental processes (Griffiths and Tenenbaum 2006; Perfors et al. 2011).

**Fig. 2:**
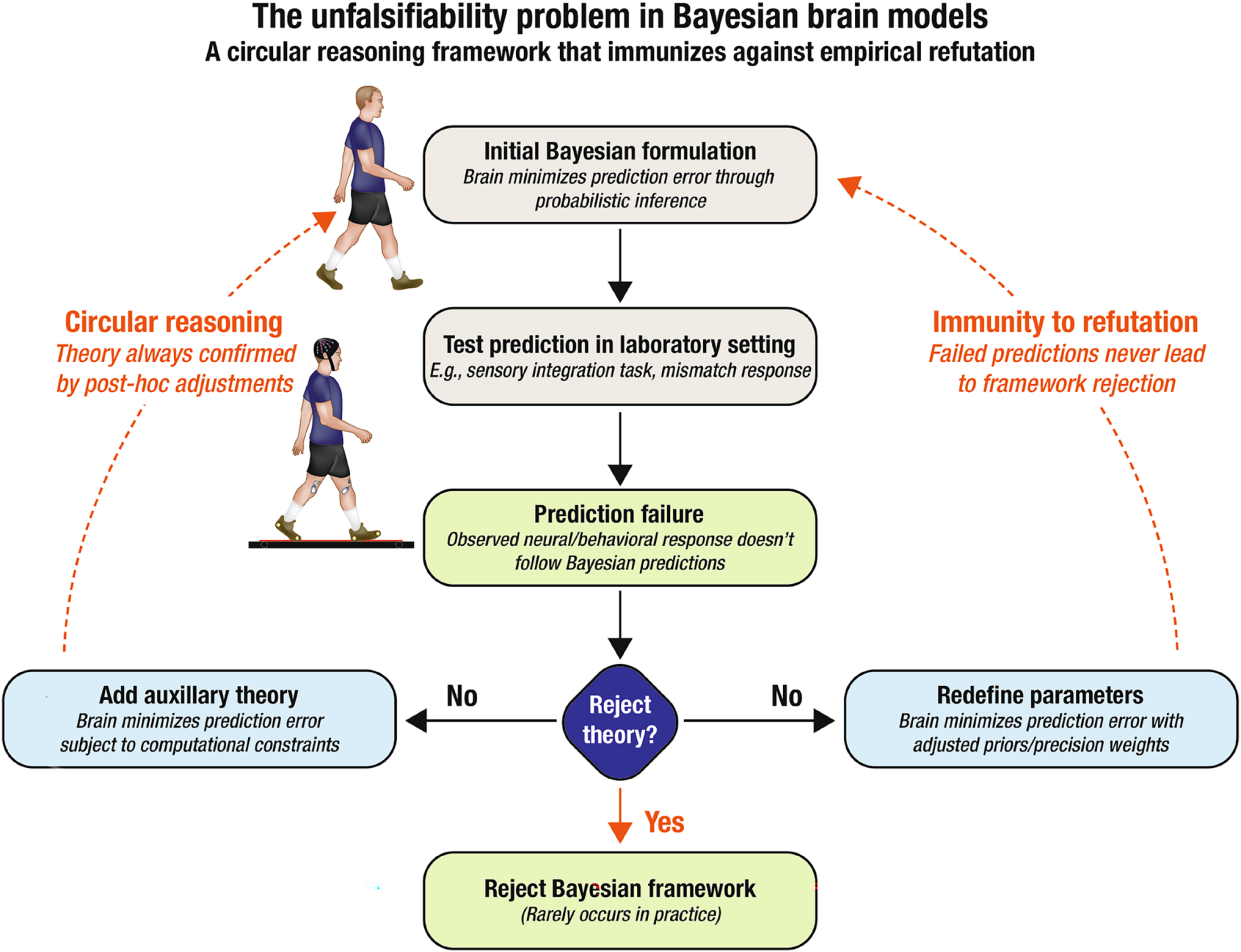
The unfalsifiability problem in Bayesian brain models. This flowchart illustrates the methodological circularity that renders the Bayesian brain hypothesis effectively unfalsifiable. When experimental evidence contradicts Bayesian predictions (e.g., “brain minimizes prediction error”), researchers rarely reject the framework. Instead, they systematically protect the Bayesian paradigm through post-hoc adjustments—either by adding auxiliary hypotheses (“subject to computational constraints”) or redefining Bayesian parameters (“adjusted priors/precision weights”). These adjustments create a self-reinforcing cycle that transforms empirical failures into theoretical successes, immunizing the framework against genuine falsification. This circular reasoning pattern reveals why Bayesian approaches persist despite repeated predictive failures: the theory automatically accommodates contradictory evidence through an endless proliferation of auxiliary hypotheses. As argued throughout this manuscript, this methodological structure violates Popperian standards of scientific testability, functioning more as mathematical rhetoric than as an empirically accountable mechanistic model of neural function

These issues connect to broader and longstanding critiques of unfalsifiability in cognitive neuroscience, particularly regarding using highly abstract, mathematically dense frameworks. The FEP, which incorporates and extends the Bayesian brain hypothesis, has been especially subject to such criticism. As Williams (2020) and Colombo and Wright (2021) have persuasively argued, the FEP’s sweeping mathematical formalism and conceptual elasticity enable it to accommodate virtually any empirical outcome, rendering it difficult—if not impossible—to falsify (**Fig**. 2).

The risk here is the unchecked proliferation of “just-so stories”—post hoc narratives crafted to explain observed phenomena without generating risky, testable predictions that could potentially disconfirm the theory (Gigerenzer 1998; Marcus and Davis 2013). While narrative explanations have their place in science, they become problematic when they create an illusion of understanding without genuine explanatory power or empirical constraint. Proponents might argue that such theoretical flexibility merely reflects the genuine complexity and adaptability of neural systems, rather than indicating any weakness in the theory itself—but this very defense illustrates the core concern: a theory that is never at risk of being wrong ultimately forfeits its status as a scientifically informative framework (Lakatos 2014).

This theoretical looseness threatens the very foundations of the scientific discipline. A framework that can explain everything, after all, explains nothing in particular (Lakatos 2014; Popper 2002). For the Bayesian brain hypothesis to progress beyond a compelling interpretive lens and establish itself as a genuinely scientific theory, it must articulate clear, testable conditions under which specific Bayesian mechanisms would be falsified—for example, by identifying neural circuits that ought to encode prediction errors and showing that their absence would undermine the theory, or by specifying experimental contexts in which non-Bayesian mechanisms demonstrably fail to approximate Bayesian inference. Many of these post hoc moves function less as genuine scientific revisions aimed at refining theoretical accuracy and more as rhetorical defense—strategic maneuvers designed to preserve the framework’s coherence and authority in the face of contradictory evidence. **Box 1** outlines some of the most common—and how to respond.

**Table:** 

Box 1: Twelve common defenses of the Bayesian brain And how to respond	
1.“You’re misusing Marr’s levels of analysis.”	
Defense: “Marr distinguished between computational and implementational levels.”	
Response: But even computational theories need implementational constraints. Otherwise, they become metaphysical abstractions.	
2. “Of course, the brain isn’t doing exact Bayesian updates.”	
Defense: “The brain uses approximations—sampling, probabilistic population codes, etc.”	
Response: Approximate how? Vague gestures toward sampling aren’t mechanisms. Show us the circuitry or concede it’s just a metaphor.	
3. “You’re ignoring all the empirical successes.”	
Defense: “Bayesian models have explained multisensory integration, illusions, and behavior.”	
Response: Most of those are post hoc curve-fitting exercises. Where are the risky predictions and independent validations?	
4. “This theory spans all of cognition.”	
Defense: “Predictive coding unifies perception, action, learning, and psychiatry.”	
Response: Theories that explain everything without constraint often explain nothing. Breadth without falsifiability is rhetorical sprawl.	
5. “It’s evolutionarily plausible.”	
Defense: “Natural selection would favor near-optimal inference strategies.”	
Response: Evolution favors robustness and speed, not statistical optimality. Metabolic efficiency beats elegance every time.	
6. “There’s neural evidence for prediction error coding.”	
Defense: “BOLD signals, EEG responses, and repetition suppression all support predictive coding.”	
Response: Correlation is not implementation. These signals are inconsistent, context-dependent, and theoretically overfitted.	
7. “We’re still refining the model.”	
Defense: “Science progresses by refining theories in light of new data.”	
Response: If every contradiction leads to a new auxiliary clause, you’re not refining—you’re shielding the theory from genuine falsification.	
8. “You’re attacking a straw man.”	
Defense: “Nobody really believes the strong version of the Bayesian brain.”	
Response: Then stop writing papers claiming the brain encodes priors, computes posteriors, and minimizes free energy.	
9. “It’s the best model we have.”	
Defense: “Bayesian models outperform alternatives in many tasks.”	
Response: Dominance in the literature may reflect institutional momentum, not epistemic superiority. Utility isn’t legitimacy.	
10. “You’re confusing levels of explanation.”	
Defense: “Computational theories don’t need neural mechanisms.”	
Response: Theories that stay permanently at the computational level become theology. At some point, they must cash out biologically.	
11. “We never said the brain explicitly computes probabilities.”	
Defense: “The brain implements functionally equivalent strategies.”	
Response: Then define what counts as equivalence—and show it with data, not wishful mappings.	
12. “Predictive coding already includes embodiment.”	
Defense: “Active inference unites brain, body, and environment.”	
Response: Only on paper. In practice, embodiment is often a bolt-on module to an inference engine, not a core architectural principle.	

## Empirical and biological Shortcomings

The brain may whisper in mathematics, but it speaks in flesh. No amount of probabilistic elegance can overcome the stubborn facts of neural tissue: its metabolic constraints, its non-Gaussian dynamics, and its embodied evolutionary history that prioritized survival over statistical optimality.

Beyond conceptual concerns, the Bayesian brain hypothesis faces substantial empirical and biological challenges that are often underappreciated in theoretical discussions.

### Neurobiological implementation challenges

First is the question of how (and whether) the brain could encode full probability distributions. Bayesian models typically operate on probability distributions over possible world states, often involving high-dimensional, continuous spaces. Representing such distributions poses significant challenges for neural systems constrained by finite resources. Researchers have proposed potential mechanisms, including population coding, sampling-based representations, and distributed encoding schemes. However, these proposals remain largely speculative and face substantial implementation challenges (Fiser et al. 2010; Pouget et al. 2013).

Second is the computational implausibility of explicit Bayesian updating in real-time. Exact Bayesian inference is computationally intensive even for simple problems and becomes intractable for complex ones. While approximate inference methods exist (variational inference, Monte Carlo sampling, etc.)—and have been proposed as biologically plausible strategies, these still require sophisticated computations that strain biological plausibility, especially for real-time decision-making under pressure (Griffiths et al. 2015; Sanborn and Chater 2016).

The temporal dynamics of neural processing present another challenge. Bayesian updating is typically formulated as a sequential process, but real neural systems operate with parallel, recurrent dynamics across multiple timescales. Reconciling these temporal patterns with idealized Bayesian updating remains problematic (Pouget et al. 2013).

### Empirical mismatches

Beyond implementation concerns, empirical evidence for Bayesian processing in the brain remains, at best, mixed and inconsistent. While some studies report behavioral patterns that align with Bayesian predictions, an equally substantial number reveal systematic deviations that question the framework’s generality. Human perception and decision-making routinely exhibit robust cognitive biases and heuristics that appear distinctly non-Bayesian, including base-rate neglect, availability bias, and anchoring effects (Kahneman and Tversky 2013; Tversky and Kahneman 1974). These deviations are not merely incidental or reducible to noise; rather, they reflect stable, reproducible patterns observed across a wide range of tasks, populations, and experimental contexts. Such findings raise important questions about whether the brain genuinely performs probabilistic inference or whether Bayesian models are retrospectively fitted to behaviors that emerge from fundamentally different, and perhaps more heuristic, cognitive mechanisms.

At the neural level, the evidence for Bayesian processing is similarly equivocal and uneven. While some studies identify neural signatures that appear to correlate with predictions derived from Bayesian models, these findings are frequently inconsistent across experimental tasks, stimulus conditions, and individual participants. Moreover, alternative non-Bayesian models—grounded in heuristics, dynamical systems, or adaptation mechanisms—can often account for the same neural patterns with equal or even greater parsimony and explanatory clarity (Rahnev and Denison 2018).

Perhaps most problematically, the Bayesian framing has difficulty accounting for the context-sensitivity and task-dependence of neural responses. The same neural circuits may perform different functions depending on behavioral context, attentional state, or motivational factors—a flexibility that sits uncomfortably with strict Bayesian formulations (Gilbert and Li 2013). These shortcomings suggest that while Bayesian principles may successfully capture certain aspects of neural processing under narrowly defined or experimentally controlled conditions, they fall short as a comprehensive or generalizable account of how the brain actually operates across the full spectrum of contexts, environments, and real-world challenges (Gigerenzer and Gaissmaier 2011; Summerfield and Tsetsos 2015).

### Revisiting the “successes:” What do Bayesian models actually explain?

Proponents of the Bayesian brain hypothesis often point to a set of well-known empirical findings—such as optimal multisensory integration, visual illusions, and motor control—as evidence of Bayesian computation in the brain. These case studies are frequently cited as successes that validate the framework and demonstrate its predictive and explanatory power (Ernst and Banks 2002; Körding and Wolpert 2004; Weiss et al. 2002). However, a closer examination reveals that these findings are often consistent with multiple theoretical interpretations. This is especially true when priors and likelihoods are fitted retroactively, require strong post hoc assumptions to align with the data, or fail to establish any clear neural implementation of Bayesian principles (Bowers and Davis 2012; Jones and Love 2011). What appears to support the hypothesis may instead reflect the descriptive flexibility of the models rather than evidence of mechanistic reality (Marcus and Davis 2013; Rasmussen and Eliasmith 2013).

**Multisensory cue integration.** A classic example comes from studies on visual-haptic integration, which report that humans combine sensory cues in a statistically optimal way, as predicted by Bayesian models (Ernst and Banks 2002). These findings are often interpreted as evidence that the brain computes a weighted average of sensory inputs based on their relative reliabilities. Yet such behavior can also emerge from experience—driven calibration or embodied strategies that do not require explicit probabilistic inference. Moreover, deviations from optimal integration have been observed in tasks with time constraints or novel stimuli (Arnold et al. 2019; Stanford et al. 2005), suggesting that the Bayesian fit may reflect task-specific tuning rather than a general cognitive strategy.

**Visual illusions.** Bayesian models have also been used to explain classic perceptual illusions, such as Adelson’s checker shadow illusion or motion illusions under ambiguous input (Knill and Richards 1996). These interpretations often posit that the brain uses prior knowledge to “explain away” unexpected sensory inputs. However, the priors in such models are typically inferred from the illusion itself—raising concerns about circular explanation. In many cases, non-Bayesian accounts based on ecological constraints or perceptual heuristics offer equally plausible, and often more parsimonious, explanations (Purves et al. 2011).

**Sensorimotor control.** In motor behavior, optimal feedback control models grounded in Bayesian inference have been widely proposed to explain how humans plan and correct movement trajectories under conditions of uncertainty (Todorov and Jordan 2002). These models have been notably successful in capturing certain features of motor behavior, such as movement efficiency, variability scaling, and adaptive responses to perturbations. However, they rely on idealized assumptions about cost functions, internal forward models, and noise distributions—assumptions that are often abstract and difficult to validate empirically. Crucially, these internal quantities are not directly measurable in biological systems and are frequently tuned post hoc to fit the observed data, rather than being specified as a priori. While this flexibility enables the models to accommodate a wide range of behavioral patterns, it substantially weakens their capacity to generate novel, falsifiable predictions about the actual neural mechanisms involved (Wolpert et al. 2011).

**Pathophysiology.** Another prominent “success story” of the Bayesian brain framework is its application to psychiatric conditions, particularly schizophrenia. Predictive coding accounts propose that symptoms such as hallucinations and delusions arise from aberrant precision-weighting of prediction errors—either attributing too much weight to low-level sensory signals or too little to top-down priors (Adams et al. 2013; Corlett et al. 2019; Friston 2016). While compelling in its post hoc coherence, this interpretation suffers from the same problems discussed throughout this paper. The models typically explain observed symptoms by adjusting the relative weight of priors or sensory inputs, but rarely predict which patients will exhibit which symptoms under specific conditions (Sterzer et al. 2018). Moreover, non-Bayesian alternatives—such as models based on disordered neuromodulation (Hu et al. 2015), disrupted synaptic pruning (Sekar et al. 2016), or abnormal cortical dynamics (Uhlhaas et al. 2009)—can also account for similar symptom profiles without invoking probabilistic inference. The Bayesian explanation, while mathematically elegant, risks functioning more as a narrative scaffold than a mechanistic theory of pathophysiology (Williams 2018). Without direct empirical markers of prediction error precision or falsifiable predictions about symptom onset or treatment response, such models offer little more than descriptive reinterpretation (Bowers and Davis 2012; Colombo 2018).

These case studies reveal a common pattern: empirical phenomena interpreted as Bayesian successes often rely on fitting data to flexible models that are agnostic about implementation. While these models may provide useful descriptive tools, but they do not necessarily demonstrate that the brain performs Bayesian inference or confirm the mechanistic claims often associated with the Bayesian brain hypothesis. A prominent example is the modeling of auditory mismatch negativity (MMN) using predictive coding frameworks. While Bayesian models can simulate MMN waveforms, they typically require finely tuned priors and assume hierarchical precision-weighting mechanisms without identifying specific neural circuits that implement them. Non-Bayesian alternatives—such as models based on local adaptation, Hebbian plasticity, or dynamic resonance—have reproduced similar MMN features without invoking hierarchical inference, raising questions about the unique explanatory value of Bayesian accounts.

## Neurobiological constraints and the implausibility of Bayesian implementation

Even the most elegant computation becomes meaningless noise when it outpaces biological possibility. The gap between theoretical Bayesian inference and actual neurophysiology is not merely a matter of approximation—it is a fundamental category error, like trying to run quantum algorithms on an abacus.

### Core assumptions of the Bayesian brain hypothesis

The Bayesian brain hypothesis rests on at least five key assumptions that warrant careful scrutiny:

1. **Representation of full probability distributions:** The framework assumes neural systems can encode and manipulate complete probability distributions over possible world states, including prior beliefs, likelihoods, and posteriors (Ma et al. 2006; Pouget et al. 2013).
2. **Real-time Bayesian updating:** It presupposes that neural circuits can perform or approximate Bayesian inference rapidly enough to support real-time perception and decision-making (Knill and Pouget 2004; Friston 2010).
3. **Hierarchical error propagation:** The predictive coding variant assumes a specific hierarchical organization where higher levels send predictions to lower levels and receive precision-weighted prediction errors in return (Rao and Ballard 1999; Friston 2005).
4. **Precision weighting:** The model assumes neural systems can dynamically adjust the “precision” (inverse variance) of prediction errors based on contextual factors and uncertainty (Feldman and Friston 2010; Hohwy 2012).
5. **Generative models:** Perhaps most fundamentally, it assumes the brain maintains internal generative models of the world that can simulate expected sensory inputs (Hinton 2007; Clark 2013).

### Non-Gaussian nature of neural fluctuations

A critical yet frequently overlooked constraint on the Bayesian brain hypothesis involves the statistical properties of neural fluctuations. Most Bayesian models implicitly or explicitly assume that neural noise conforms to Gaussian (normal) distributions—a choice made more for mathematical tractability than for biological realism. However, a growing body of empirical evidence indicates that neural activity consistently exhibits distinctly non-Gaussian statistical properties across multiple temporal and spatial scales of brain organization:

1. **Heavy-tailed distributions:** Neural fluctuations frequently follow heavy-tailed distributions, including power-law, log-normal, and Lévy-stable distributions rather than Gaussian profiles (Beggs and Plenz 2003; Chialvo 2010; Freyer et al. 2009; Metzler and Klafter 2000; Touboul and Destexhe 2010). Such distributions imply that large-amplitude events occur much more frequently than would be expected under Gaussian assumptions, fundamentally altering the statistical landscape within which neural computation must operate.
2. **Scale-free dynamics:** The power spectra of neural signals typically follow a power-law form ($1/f^{\beta }$), indicating temporal correlations that persist across multiple timescales (He et al. 2010; Miller et al. 2009; Linkenkaer-Hansen et al. 2001; Touboul and Destexhe 2010). This scale-free property contradicts the assumption of temporally uncorrelated noise that underlies many Bayesian formulations.
3. **Bistability and multistability:** Neural activity often exhibits multimodal distributions, reflecting transitions between distinct dynamical states rather than continuous fluctuations around a single equilibrium (Deco et al. 2011; Freyer et al. 2009; Kinouchi and Copelli 2006). Such bistable or multistable dynamics cannot be adequately captured by conventional Bayesian frameworks that presume unimodal posterior distributions.
4. **Multiplicative noise processes:** Evidence suggests that neural fluctuations involve multiplicative rather than merely additive noise components, where the variance of the noise depends on the system’s state (Buice et al. 2010; Roberts et al. 2019). These multiplicative interactions create fundamentally different statistical properties than the additive noise typically assumed in Bayesian models.

These non-Gaussian characteristics of neural fluctuations present severe challenges to the Bayesian brain hypothesis, suggesting that the simplifying assumptions underlying many Bayesian models may systematically misrepresent the true nature of brain function. Conventional Bayesian inference becomes analytically intractable under non-Gaussian noise conditions, and approximation methods often fail to capture the rich statistical structure of real neural activity. While proponents might argue that the brain could implement specialized algorithms to perform Bayesian inference under non-Gaussian conditions, such implementations would require vastly more computational resources than standard Bayesian methods and would introduce additional layers of complexity that strain biological plausibility.

At the same time, the widespread presence of non-Gaussian statistics in neural systems suggests that the brain may rely on fundamentally different computational principles than those assumed by standard Bayesian theory. Dynamic systems approaches, which naturally account for non-Gaussian fluctuations, criticality, and multistability, offer a compelling alternative. These frameworks may provide more biologically realistic accounts of neural computation than the idealized models of probabilistic inference central to the Bayesian brain hypothesis.

### Neuroanatomical inconsistencies: brain structures and circuit properties incompatible with Bayesian processing

The Bayesian brain hypothesis faces not only abstract computational challenges but also concrete neuroanatomical inconsistencies that fundamentally undermine its biological plausibility. These structural and functional properties of real neural tissue present obstacles that no amount of mathematical elegance can overcome:

- **Dendritic integration dynamics:** Contrary to the precise weighted summation required by Bayesian models, dendritic integration in actual neurons exhibits highly nonlinear, context-dependent properties. Dendrites contain voltage-gated ion channels that create complex integration zones with supralinear and sublinear summation depending on the spatiotemporal pattern of inputs (Branco and Häusser 2010; London and Häusser 2005; Polsky et al. 2004). These dynamics create input–output functions that cannot be mapped onto the linear weighted averaging required for combining likelihoods and priors in Bayesian calculations. Moreover, single neurons often exhibit branch-specific plasticity rather than global weighting adjustments (Losonczy et al. 2008), contradicting the assumption that synaptic weights represent statistical parameters in a coherent probabilistic model.
- **Neuromodulatory effects on circuit dynamics:** Bayesian frameworks typically assume stable computational mechanisms with adjustable parameters (e.g., precision weighting). However, neuromodulators like dopamine, acetylcholine, and norepinephrine fundamentally reconfigure circuit properties—not merely adjusting weights but qualitatively changing input–output functions, intrinsic excitability, and even the direction of plasticity (Dayan 2012; Marder et al. 2014). This creates context-dependent processing modes that cannot be characterized as mere parameter adjustments within a stable Bayesian architecture. The pervasive influence of these neuromodulatory systems means that neural circuits are constantly switching between fundamentally different computational regimes—an observation incompatible with the consistent application of Bayesian principles.
- **Recurrent connectivity patterns and attractor dynamics:** Cortical and subcortical circuits exhibit dense recurrent connectivity that generates attractor dynamics, oscillatory patterns, and metastable states (Brinkman et al. 2022; Deco and Hugues 2012). These dynamics create temporal dependencies that violate the Markov assumptions underlying sequential Bayesian updating. Furthermore, the prevalence of inhibitory interneurons creates winner-take-all dynamics and competitive inhibition that force categorical rather than probabilistic representations—a pattern seen in the sharp tuning curves and bistable activity patterns observed across cortical regions (Buzsáki 2006; Isaacson and Scanziani 2011). These patterns indicate that neural circuits are organized around attractor dynamics rather than the continuous probability distributions required for Bayesian processing.
- **Sparse, synchronous coding schemes:** Bayesian models typically rely on distributed representations to encode full probability distributions across high-dimensional spaces. In contrast, empirical evidence suggests that actual neural activity is highly sparse and temporally structured. Many brain regions exhibit population activity where only $< 10\%$ of neurons are active simultaneously, with spike timing locked to ongoing oscillatory rhythms (Fries 2015; Olshausen and Field 2004). While sparse coding is metabolically efficient and well-suited for feature extraction, it poses challenges for implementations that require continuous probabilistic sampling or integration across neural populations. The temporal and anatomical constraints that give rise to sparse, synchronous activity are not just computational limitations—they reflect architectural principles that may be fundamentally incompatible with the assumptions of Bayesian inference. Encoding and maintaining full posterior distributions in such systems would require coordination and representational bandwidth not supported by current neurophysiological data (Olshausen and Field 2004). Thus, the mismatch is not merely one of degree, but of representational format and underlying biophysical feasibility.
- **Anatomical specialization versus domain-general Bayesian processing:** The brain exhibits extreme anatomical specialization with distinct cell types, circuit motifs, and processing principles across regions—from the unique neuronal types of the cerebellum to the columnar organization of sensory cortices to the distinct microcircuits of the basal ganglia (Harris and Shepherd 2015; Nelken 2004). This neuroanatomical diversity contradicts the assumption of a uniform Bayesian processing principle operating throughout the brain. If Bayesian inference were a core organizing principle, we would expect greater uniformity in computational architecture; instead, we observe region-specific wiring patterns optimized for particular transformations rather than general-purpose probabilistic inference.
- **Developmental constraints on precision-based hierarchies:** Predictive coding models within the Bayesian brain framework assume hierarchically organized precision-weighting mechanisms. However, examining the developmental trajectory of neural circuits reveals that these supposedly precision-weighted connections develop according to genetically guided principles and activity-dependent pruning mechanisms that do not incorporate statistical precision information (Katz and Shatz 1996; Wong and Ghosh 2002). The laminar connections that supposedly implement hierarchical message-passing develop through molecular guidance cues and critical period mechanisms that are insensitive to the statistical properties predictive coding requires. These developmental constraints produce circuit architectures determined more by embryological and molecular factors than by the statistical requirements of Bayesian inference.
- **Non-parametric plasticity mechanisms:** Synaptic plasticity mechanisms—the presumed basis for learning statistical relationships in Bayesian models—operate according to principles incompatible with Bayesian updating. Long-term potentiation and depression depend on coincidence detection, postsynaptic calcium levels, retrograde messengers, and structural reorganization rather than error-based updating of statistical parameters (Feldman 2012; Malenka and Bear 2004). These mechanisms produce binary-like, threshold-dependent changes rather than the continuous parameter adjustments required for proper Bayesian updating. Furthermore, homeostatic plasticity operates to maintain overall activity levels rather than statistical accuracy (Turrigiano and Nelson 2004), often pushing synaptic weights in directions opposite to what Bayesian learning would predict.

These neuroanatomical inconsistencies collectively present insurmountable challenges to any literal interpretation of the Bayesian brain hypothesis. The fundamental organizing principles of neural tissue—from dendritic integration to circuit motifs to plasticity mechanisms—reflect evolutionary pressures favoring robustness and energy efficiency under biological constraints, rather than the implementation of probabilistic inference. While mathematical models might abstract away these biological details, a truly scientific theory of brain function cannot ignore the actual properties of the substrate it purports to explain. The mismatch between Bayesian computational requirements and neuroanatomical reality suggests that the brain employs fundamentally different organizational principles than those proposed by the Bayesian framework. If the theory demands mechanisms that biology cannot provide, then the elegance of its math is no substitute for empirical viability.

### Neurophysiological implausibility

These assumptions face significant challenges when confronted with known properties of neural systems:

1. **Metabolic and computational constraints:** Exact Bayesian inference is computationally intensive. While approximate methods exist, even these impose substantial burdens that appear incompatible with the brain’s energy budget. Neural tissue is metabolically expensive, consuming approximately 20% of the body’s energy despite comprising only 2% of body mass (Attwell and Laughlin 2001; Magistretti and Allaman 2015). The computational resources required for full Bayesian inference across multiple sensory modalities would impose prohibitive energy costs (Harris et al. 2012).
2. **Temporal dynamics mismatch:** Neural processing occurs through complex parallel and recurrent dynamics across multiple timescales. These dynamics do not align well with the sequential, iterative nature of Bayesian updating. The brain must respond to environmental challenges within milliseconds, while proper Bayesian inference in complex state spaces would require extensive computation time, creating a fundamental temporal mismatch (Csicsvari et al. 1999; Hari and Parkkonen 2015).
3. **Representational capacity limitations:** Representing full probability distributions over high-dimensional state spaces would require vast neural resources. Simple laboratory tasks might involve only a few variables, but real-world perception involves thousands of interacting dimensions. The representational capacity required exceeds what could plausibly be implemented in neural tissue, particularly for continuous variables with theoretically infinite possible states (Beck et al. 2012; Fiser et al. 2010).
4. **Noise and variability:** Neural systems exhibit substantial intrinsic noise and variability at all levels, from ion channel fluctuations to large-scale network dynamics (Renart et al. 2010). While Bayesian models elegantly incorporate uncertainty, they typically assume well-behaved noise distributions that may not match the complex, state-dependent noise characteristics of real neural systems. This intrinsic neural variability would systematically undermine the precision of any attempted Bayesian computations.
5. **Context-dependent processing:** Neural responses show remarkable context-sensitivity, with the same circuits serving different roles in processing different behavioral states, neuromodulatory influences, and task demands (Bargmann 2012; Gilbert and Li 2013). This flexible, adaptive processing conflicts with the relatively rigid computational structure implied by hierarchical Bayesian models. Furthermore, neuromodulatory systems dynamically reconfigure circuit properties in ways that do not map cleanly onto Bayesian parameters like precision-weighting.
6. **Implementation gap:** Proponents have suggested various neural implementations of Bayesian computations, including population coding, probabilistic population codes, and sampling-based approaches (Pouget et al. 2013). However, these proposals remain largely theoretical and lack direct empirical confirmation. The neural mechanisms that could perform the necessary computations—encoding priors, computing likelihoods, and integrating these according to Bayes’ rule—have not been convincingly shown in biological neural circuits.
7. **Developmental implausibility:** The Bayesian framework struggles to explain how appropriate priors and generative models could be established during development. While some priors might be genetically encoded, many must be learned through experience. This creates a bootstrapping problem: how could a developing brain learn appropriate probabilistic models without sufficient models to interpret its experiences? (Smith and Gasser 2005; Zhao et al. 2024)
8. **Evolutionary considerations:** From an evolutionary perspective, organisms require fast, efficient, and reliable mechanisms that are sufficiently effective for survival in complex and often unpredictable environments—not mechanisms that necessarily deliver statistically optimal inference. Natural selection tends to favor strategies that work well enough under real-world constraints, including limited time, energy, and computational capacity. The high computational complexity and resource demands of full Bayesian inference make it an implausible candidate for biological implementation, especially when simpler, more robust heuristic strategies can yield adaptive behavior with far less cost. These heuristics, shaped by evolutionary pressures, often perform remarkably well in natural settings despite their departure from statistical optimality (Cosmides and Tooby 1996; Gigerenzer and Goldstein 1996; Gigerenzer and Brighton 2009; Gigerenzer 2020; Marewski and Schooler 2011). As such, the brain is more likely to implement these satisficing solutions than to engage in the exhaustive computations required by normative Bayesian models.

These neurobiological constraints collectively suggest that the Bayesian brain hypothesis, while mathematically elegant, faces serious limitations as a literal account of neural function. Although certain behaviors may resemble Bayesian inference in controlled settings, the underlying neural mechanisms likely rely on non-Bayesian, heuristic processes that are more consistent with the biological realities of the brain. Neural systems operate under severe constraints—metabolic, temporal, and structural—that often preclude the kind of optimal computation the hypothesis assumes. Despite these challenges, the Bayesian framework continues to shape theoretical models, sometimes more for its conceptual appeal than its empirical plausibility. This enduring influence underscores the need for deeper scrutiny of its assumptions and a greater willingness to engage with alternative models better suited to the messy, adaptive nature of living systems.

The limitations of the Bayesian brain hypothesis become particularly apparent when compared to alternative theoretical frameworks that conceptualize cognition in fundamentally different terms. These alternatives offer not just competing explanations but challenge the basic assumptions upon which the Bayesian approach rests.

## Alternative views of cognition

To know a mind, you must first acknowledge that it evolved not in the pristine realm of probability distributions, but in the messy, constraint-laden world of bodies navigating environments. Cognition emerges not from internal models but from the dynamic dance between organism and world.

The limitations of the Bayesian brain hypothesis become clearer when contrasted with alternative frameworks that conceptualize cognition in fundamentally different terms. These alternatives do not merely offer competing accounts within the same basic paradigm but challenge the foundational assumptions on which Bayesian models rest. Where Bayesian models begin with inference, representation, and error minimization, these alternatives begin with embodiment, interaction, and dynamic constraint—treating cognition not as a computational problem solved by internal models but as an emergent phenomenon of systems embedded in material, temporal, and relational contexts, shaped continuously by bodily dynamics, environmental affordances, and the irreversible flow of lived experience.

### Dynamic systems approaches

Dynamic systems theory considers cognition emerging from complex, nonlinear interactions among neural, bodily, and environmental components (Kelso 1995; Thelen and Smith 1994; Van Orden et al. 2003). Rather than focusing on information processing or probabilistic inference, this approach emphasizes how cognitive functions self-organize through the continuous coordination of processes unfolding across multiple timescales (Beer 2000; Sporns and Edelman 1993). Prediction, in this framework, is not the outcome of internal model selection but a natural consequence of temporally structured dynamics—where the system’s history and current state constrain its future evolution, without requiring explicit Bayesian updating (Schmidt and Richardson 2008; Richardson et al. 2014).

### Ecological psychology

The ecological approach rejects the assumption that perception requires inference, reconstruction, or internal models of the world (Gibson 1966, 1979). Instead, it proposes that organisms directly perceive affordances—possibilities for action—by detecting invariant structures in ambient sensory arrays (Chemero 2009; Turvey 1992). Perception, then, is not a problem of interpretation under uncertainty but a continuous process of attunement to ecological information. It is fundamentally action-oriented, relational, and environment-coupled, grounded in real-time interaction rather than mediated by internal representations or probabilistic inference (Michaels and Carello 1981; Warren 2021). This orientation stands in direct contrast to Bayesian frameworks, which assume that perception involves constructing internal hypotheses about hidden causes of sensory input; the ecological view holds that no such detour through representation is necessary when information is already available in the structure of the environment.

### Embodied and enactive cognition

Embodied cognition emphasizes how cognitive processes are shaped by the physical body and its sensorimotor interactions with the world (Clark 1999; Shapiro 2019; Varela et al. 1991). Related enactive approaches view cognition as constituted by the dynamic coupling between organism and environment, rather than as computation occurring within the brain (Di Paolo et al. 2017; Thompson and Varela 2001). These perspectives shift the explanatory focus from internal models to the organism-environment system as the proper unit of analysis (Gallagher 2017; Noë 2004). What unites these alternatives is their treatment of cognition as emergent, context-sensitive, and fundamentally non-inferential. They suggest that prediction and adaptive behavior can arise from rich dynamical coupling with the environment, without necessarily involving probabilistic inference or Bayesian computation (Anderson 2014; Chemero 2009).

Importantly, these frameworks offer empirically distinct predictions from Bayesian models. For instance, they predict that cognitive performance will be highly sensitive to bodily states and environmental contexts in ways that purely brain-based inferential models might not anticipate (Barsalou 2008; Spivey 2007). They also suggest that the temporal dynamics of neural activity may reflect ongoing engagement with the environment rather than sequential updating of internal models (Kiverstein and Rietveld 2018; Raja 2019).

By taking these alternatives seriously, we gain a broader and more grounded perspective on the limitations of the Bayesian brain hypothesis. What appears from one theoretical vantage point as probabilistic inference may, from another, be more coherently understood as dynamic coupling, direct perception, or embodied action unfolding in real time. These frameworks do not merely shift explanatory emphasis; they pose a conceptual alternative, though their ability to generate tightly constrained predictions remains an open challenge. This pluralistic approach reminds us that mathematical descriptions, however elegant or internally consistent, represent only one possible way of carving nature at its joints—and that the joints themselves may not always lie where the equations suggest (Dale et al. 2013; Richardson et al. 2008). By loosening the grip of inference and embracing interaction, these alternatives invite a neuroscience more attuned to the realities of living systems than to the ideals of formal logic.

A full comparative evaluation of these alternative frameworks is beyond the scope of this critique, which is focused on exposing the limitations of the Bayesian paradigm. However, we acknowledge the importance of more thoroughly articulating the empirical and mechanistic commitments of these approaches, and we view that effort as a necessary complement to the present work. Such work will require not only detailing their successes but also clarifying the domains in which they outperform—or meaningfully diverge from—Bayesian accounts.

## A fictional dialogue: Critic vs. Bayesian theorist

The more universal a theory claims to be, the more invisible its assumptions become. What appears as mathematical insight may simply be metaphysical sleight-of-hand—turning epistemic flexibility into evidence, and unfalsifiability into strength.

To illustrate the core conceptual tension at the heart of the Bayesian brain and Free Energy Principle frameworks, we present a fictional but representative exchange between a critical interlocutor and a proponent (though this dialogue does not reproduce actual statements). This constructed dialogue serves as a heuristic device to clarify where the theory’s strengths intersect with its vulnerabilities—particularly in terms of scope, testability, and mechanistic specificity. It is not intended as a verbatim transcript but as a distillation of recurring arguments on both sides.

**Critic:** Dr. Bayesian theorist, you’ve argued that the Free Energy Principle (FEP) underlies all self-organizing systems, from bacteria to candle flames, and that the brain operates in a fundamentally Bayesian way—constantly minimizing “surprise.” Critics worry this makes your theory unfalsifiable. They say, “If *everything* is doing Bayesian inference, the idea loses empirical bite.” How do you respond?

**Bayesian theorist:** The principle *is* universal: any system maintaining its integrity—bacterium, cell, brain—can be cast as minimizing free energy. I don’t see that as a drawback. Universality reflects the fundamental nature of self-organization, *not* a lack of empirical content.

**Critic:** But critics point to your own examples: a *candle flame* or a *bacterium* apparently also “minimizes free energy.” Yet no one claims a flame is *actually computing* Bayesian posteriors. Doesn’t that ring of absurdity?

**Bayesian theorist:** To call it “absurd” is to miss the point. The candle-flame example shows a physical system evolving in a way that *can* be described using the same mathematics we apply to living systems. That doesn’t mean the flame has internal generative models. It means the underlying dynamics—entropy flows, thermodynamics—are expressible in free-energy terms. The same math can describe everything from quantum phenomena to adaptive brains.

**Critic:** That’s exactly the problem: a flame is obviously *not* thinking or updating priors. If *both* nonthinking flames *and* sophisticated brains are described by “Bayesian” or “free-energy” equations, it looks like you’re labeling *any* self-organizing process “Bayesian.”

**Bayesian theorist:** You could call it Bayesian in a formal sense, but I see it as evidence of a unifying principle. The brain’s *internal dynamics* instantiate more complex versions of the same fundamental process. Don’t conflate “Bayesian explanation” with “deliberate calculation.” My point has always been that living systems *must* reduce surprise to persist, and those dynamics can be cast in Bayesian terms.

**Critic:** But if you can cast *anything* in Bayesian terms, how can we show it’s *wrong*? Couldn’t you just rewrite the model’s “priors” every time something unexpected happens, so you can say, “Look, it was always Bayesian!”

**Bayesian theorist:** The principle is not a matter of rewriting at whim. It *constrains* how you adjust priors or likelihoods. If a system *consistently* defies Bayesian predictions—for instance, if hierarchical error-correction utterly fails—we’d need a different explanation. The fact we can keep refining a Bayesian model doesn’t mean we can’t fail; it just means the FEP is broad enough to generate models for diverse scenarios.

**Critic:** In everyday neuroscience, illusions, multisensory integration, and motor control are called “Bayesian success stories.” Skeptics say those models have so many parameters that even odd results can be fitted retroactively. Where’s the big falsification? Couldn’t a sufficiently flexible Bayesian model *also* explain me walking into a lamppost?

**Bayesian theorist:** (Laughs) Overfitting can happen in any modeling framework. But we focus on *mechanistic detail*—which neural populations encode priors, how synapses implement error-correction, and so on. If we cannot locate or measure these processes in *actual* neural data, then the model remains purely formal. But real Bayesian neuroscience tries to ground the math in genuine physiology.

**Critic:** Yet your own candle-flame or bacterial examples seem to trivialize that. They also show up in discussions of the Free Energy Principle: a flame or a microbe doesn’t have neurons, so how is it *Bayesian*? Some observers see that as a reductio ad absurdum.

**Bayesian theorist:** I never claimed a flame *literally* performs Bayesian updates. It’s about self-organization—the math is universal, but the specific *mechanistic* implementation differs vastly. A candle flame has only a transient boundary, while an organism has a stable Markov blanket that supports hierarchical processes akin to Bayesian inference.

**Critic:** Still, you give an impression that *everything* fits under free-energy minimization, so critics wonder if it’s truly a *testable* theory. Doesn’t its sweeping scope risk nonfalsifiability?

**Bayesian theorist:** People conflate broad applicability with an absence of testability. The principle is *broad*, but actual *models* of neural systems are specific. Take predictive coding in vision, for example: if certain error signals or top-down predictions aren’t found where the theory says they should be, that challenges the model. Some of these concerns are valid—especially when Bayesian models are used metaphorically or post hoc. But well-specified models can and do make falsifiable predictions.

**Critic:** And if the data don’t match, you can just posit new priors or updated likelihoods. That’s what *makes* it feel slippery. Maybe the candle flame is a comedic extreme, but to many, it exemplifies how you can “explain” anything by shoehorning it into a Bayesian or free-energy story.

**Bayesian theorist:** I accept that critics see it that way. My stance is that the theory’s generality shows how universal processes, like thermodynamic minimization, continue into biological and cognitive realms. If you want to call that “slippery,” so be it. I view it as a deep unification of physics and biology. Whether one finds that profound or suspicious might depend on one’s philosophical commitments.

**Critic** *(turning to the audience):* So there you have it. Dr. Bayesian theorist maintains the view that the Free Energy Principle and Bayesian frameworks are universal, and that this universality doesn’t negate testability. Many of us remain skeptical, especially when a *candle flame* can be dubbed a free-energy minimizer. But Dr. Bayesian theorist stands firm: the math unifies how living systems arise from physical processes, and our criticisms do not bring down his core claim.

## Why the myth persists

Ideas do not spread by truth alone, but by prestige, mathematical fluency, and the comforting promise of order amid chaos. The Bayesian brain persists not because it accurately describes neural function, but because it satisfies our desire for elegant theories that render the world comprehensible through equations.

Despite its conceptual and empirical shortcomings, the Bayesian brain hypothesis continues to exert enormous influence on cognitive neuroscience. Understanding this persistence requires examining both intellectual and sociological factors that sustain the approach despite its problems. The resilience of this framework in the face of substantial criticism represents a fascinating case study in the sociology of scientific knowledge and the dynamics of theoretical entrenchment in contemporary neuroscience.

### The appeal of mathematical elegance

Bayesian models offer something precious in science: mathematical precision and formal elegance. The ability to express complex cognitive phenomena in the language of probability theory creates a satisfying sense of clarity and rigor. This mathematical tractability facilitates modeling, hypothesis testing, and theoretical integration in ways that less formalized approaches cannot match. As Kuhn (1962) noted in his analysis of scientific paradigms, the aesthetic appeal of theories—their simplicity, coherence, and mathematical beauty—often plays a crucial role in their acceptance and persistence. The Bayesian framework exemplifies this phenomenon, offering researchers a clean, formalized language that promises to bring order to the messy complexity of neural systems. This aesthetic allure is not merely a philosophical curiosity; it has real consequences for how theories gain traction and guide empirical investigations in contemporary neuroscience.

The mathematical formalism of Bayesian approaches has particular appeal in an era when neuroscience increasingly values computational sophistication (Kriegeskorte and Douglas 2018). Complex statistical techniques, computational models, and mathematical abstractions carry significant prestige in contemporary science, often serving as markers of theoretical rigor and sophistication. This “mathematization” of neuroscience reflects broader trends across the sciences, where quantitative approaches tend to displace qualitative ones, regardless of their explanatory adequacy (Gigerenzer and Brighton 2009; Gigerenzer 2020). As a result, theories are often judged more by their formal consistency and technical elegance than by their capacity to generate novel, testable insights about biological systems. The Bayesian brain hypothesis benefits from this cultural privileging of mathematical formalism, gaining credibility partly through its quantitative sophistication rather than its empirical or mechanistic plausibility.

Moreover, the Bayesian framework connects to a venerable tradition of understanding cognition as rational or optimal within constraints (Anderson 2013; Chater and Oaksford 1999). It offers a comforting vision of the brain as fundamentally rational, implementing statistically optimal solutions to cognitive problems. This aligns with longstanding philosophical and scientific aspirations to discover underlying rationality in human thought, a project that extends back through the history of cognitive science to the Enlightenment ideal of human rationality (Gigerenzer and Goldstein 1996). By echoing these deep-rooted ideals, the Bayesian approach inherits not just intellectual appeal but also a kind of cultural legitimacy that can make it resistant to empirical challenge. The Bayesian view thus appeals not only to technical considerations but to deeper cultural narratives about the nature of mind and its relationship to mathematical order.

The technical advantages of Bayesian methods further reinforce their appeal. Their capacity to handle uncertainty, incorporate prior knowledge, and update beliefs based on evidence makes them effective for modeling complex systems (Gelman et al. 1995; MacKay 2003). This utility incentivizes researchers to adopt the Bayesian framework, independent of its deeper theoretical claims about neural function. However, technical utility alone can foster a kind of theoretical complacency, where successful applications are mistaken for confirmation of foundational assumptions. As researchers invest time and resources in developing Bayesian models and methods, this momentum creates a path dependency that sustains the framework even when its foundational assumptions remain questionable.

### The power of metaphor

As a metaphor, the Bayesian brain has undeniable power. The image of the brain as a prediction machine, constantly testing hypotheses against incoming evidence, provides an intuitive and generative way of thinking about neural function (Clark 2013; Hohwy 2013). Even if the implementation details remain murky, the metaphor helps organize diverse findings and suggests new research directions. Cognitive scientists have long recognized the importance of metaphors in scientific theorizing—from computer models of mind to neural networks to dynamical systems (Gentner and Grudin 1985; Lakoff and Johnson 1980). These metaphors function not merely as pedagogical devices but as conceptual scaffolds that structure research programs and shape theoretical intuitions.

The predictive processing metaphor inherits the cultural authority of Bayesian statistics while connecting to intuitive notions of prediction and error-correction that resonate across multiple levels of description (Clark 2015). Its conceptual versatility allows it to be deployed across domains—from low-level sensory processing to high-level cognition, from individual neurons to large-scale networks. This cross-domain applicability creates a sense of theoretical unification that is deeply appealing in a field often characterized by fragmentation and specialization (Friston 2010).

The very ambiguity discussed earlier—the slippage between metaphor and mechanism—may paradoxically contribute to the framework’s appeal. This conceptual flexibility allows researchers to engage with the framework at different levels of commitment, from loose inspiration to strong mechanistic claims, depending on their goals and disciplinary backgrounds (Jones and Love 2011). Philosophers can explore its epistemological implications; computational neuroscientists can develop formal models; experimental neuroscientists can interpret neural data within its conceptual framework—all without necessarily agreeing on the precise status or scope of the claims being made. This interpretive flexibility functions as a form of “boundary object” in science studies terms—a conceptual entity that is plastic enough to adapt to local needs while robust enough to maintain a common identity across contexts (Star and Griesemer 1989). But while this flexibility facilitates interdisciplinary dialogue and theoretical innovation, it also blurs the lines between scientific theory and rhetorical scaffold, making it increasingly difficult to discern where genuine empirical content ends and metaphorical or programmatic resonance begins.

Furthermore, the predictive processing narrative offers an appealing synthesis of seemingly opposing traditions in cognitive science—bringing together representationalist and embodied approaches, computational and dynamical perspectives, and internalist and externalist views of mind (Allen and Friston 2018; Clark 2015). This synthetic quality positions the framework as a potential bridge across entrenched theoretical divides, enhancing its attractiveness to researchers from various disciplinary and philosophical backgrounds. Proponents frequently underscore this potential, suggesting that predictive processing can reconcile conflicting paradigms and move the field beyond dichotomous thinking, resolving longstanding debates rather than merely choosing sides in them (Kirchhoff 2018).

The Bayesian brain metaphor, while powerful, carries potential epistemological risks. As Kuhn (1962) noted, dominant metaphors can hinder scientific progress when they obscure observations that challenge the prevailing framework. When a metaphor transitions from productive heuristic to unquestioned background assumption, it risks becoming what Bachelard (2002) called an “epistemological obstacle”—a conceptual barrier that hinders rather than facilitates discovery. In the case of the Bayesian brain, the predictive metaphor’s very success may lead researchers to overlook or reinterpret evidence inconsistent with its basic premises, potentially obscuring alternative conceptualizations of neural function that might better account for certain aspects of cognition and behavior (Anderson 2017). This concern invites a critical question for cognitive neuroscience: not merely whether the metaphor is useful, but under what conditions should be refined, limited, or even replaced by more empirically grounded frameworks.

### Institutional and sociological factors

Scientific practices are shaped by evidence and social and institutional factors that influence research funding, publication, and recognition (Latour 1987; Longino 2020). The Bayesian brain hypothesis emerged during a period of increasing mathematization in neuroscience and psychology, when computational approaches gained prestige and institutional support (Dupré 1993; Piccinini and Craver 2011). This timing positioned it to benefit from broader trends in funding and publication, where mathematically sophisticated approaches often receive preferential treatment.

The framework has been championed by influential researchers at prestigious institutions, whose intellectual authority lends credibility and accelerates its diffusion through influential scientific networks (Whitley 2000). High-profile publications in elite journals have further reinforced its position as a dominant, mainstream approach, creating a self-reinforcing cycle in which early prominence leads to increased visibility, higher citation counts, and sustained influence across disciplines. This well-documented Matthew effect in scientific recognition—where initial advantages accumulate and intensify over time—helps explain how certain theoretical frameworks can rise to prominence and maintain dominance, even when their empirical adequacy remains contested or incomplete (Merton 1968).

Once established as a dominant paradigm, such frameworks tend to perpetuate through various mechanisms: funding agencies prioritize research within established frameworks; graduate students are trained in these approaches; journals favor papers that extend or apply the dominant paradigm. Kuhn (1962) described this phenomenon decades ago as the self-reinforcing nature of scientific paradigms, where normal science operates within established conceptual frameworks rather than challenging their foundations. The Bayesian brain has achieved paradigmatic status in certain areas of cognitive neuroscience, with researchers more likely to work within its assumptions than to question them fundamentally.

Career incentives further reinforce this pattern. Young researchers face strong pressures to publish in high-impact journals and secure competitive grants—goals more easily achieved by working within established frameworks than by challenging them (Smaldino and McElreath 2016). The safer career path involves extending or applying the Bayesian framework rather than developing alternatives or exposing its limitations, thereby creating a selection pressure in the academic ecosystem that favors theoretical conformity over innovation or critique (Akerlof and Michaillat 2018). Furthermore, the publishing landscape in contemporary science rewards novel, positive findings over replications or negative results (Ioannidis 2005; Romero 2017).

This creates pressure to adapt research to fit popular frameworks, even when the evidence is ambiguous. The flexibility of Bayesian models makes them particularly susceptible to this dynamic, as priors, precision-weighting, and hierarchical depth can be adjusted to accommodate almost any finding. Researchers have strong incentives to interpret their results as supporting or extending the Bayesian framework, rather than challenging it—leading to a literature that systematically over-represents supportive evidence (Ioannidis 2008; Nosek et al. 2012).

The interdisciplinary nature of the Bayesian brain hypothesis also contributes to its resilience. Criticisms about non-Gaussian neural dynamics from neurophysiologists can be deflected by computational theorists highlighting the framework’s mathematical elegance, while concerns about mechanistic implementation from cellular neuroscientists can be dismissed by philosophers emphasizing its unifying conceptual power (Longino 2020). These factors combine to create a situation in which the explanatory limitations of the Bayesian brain hypothesis are systematically underexplored or downplayed, while its perceived successes are prominently highlighted and widely celebrated. The result is acquiring the status of an entrenched but under-specified paradigm—a compelling narrative that endures not only because of selective empirical support, but also because of its mathematical elegance, metaphorical resonance, and accumulated sociological momentum (Smaldino and McElreath 2016). Gaining a clear understanding of these extra-empirical influences is essential for critically evaluating the Bayesian brain hypothesis’s current status and advancing more epistemically rigorous and scientifically accountable approaches to theoretical neuroscience.

As the philosopher of science Lakatos (2014) argued, research programs that continually accommodate counterevidence by adjusting auxiliary assumptions may become degenerative rather than progressive. The Bayesian brain hypothesis risks following this trajectory when confronted with mechanistic challenges—by retreating to increasingly abstract formulations of “approximated” inference, “hierarchical” cortical implementations, or “implicit” Bayesian operations that become progressively detached from testable neural mechanisms (Bowers and Davis 2012). The framework’s persistence despite substantial criticism illustrates broader patterns in scientific theorizing—how aesthetic, practical, and social factors shape theory choice alongside empirical considerations.

This does not mean that the Bayesian brain hypothesis lacks scientific value, but rather that its dominance cannot be explained by its empirical or explanatory successes alone. A full understanding of its place in contemporary neuroscience requires attention to both its intellectual content and its sociological context (Firestein 2012; Longino 2020). One key to its resilience lies in traversing different explanatory levels, often without explicitly committing to any of them. This strategic slippage is often hidden behind invocations of Marr’s framework, which, rather than clarifying explanatory levels, has become a rhetorical escape hatch. As shown in **Table** 4, this strategic slippage between Marr ’s (1982) levels of analysis allows proponents to evade empirical accountability without relinquishing theoretical authority.

**Table 4:** Levels of analysis or levels of evasion? A comparison of how Marr’s levels are invoked in ideal theory versus how they function rhetorically within Bayesian brain literature

Level (Marr, 1982)	Original purpose	Bayesian brain rhetorical use
**Computational**	Define the problem the system solves and why	Used to justify abstract probabilistic goals (e.g., inference, minimization) without committing to implementational details
**Algorithmic**	Specify the process and representations used	Often skipped or loosely sketched; Bayesian updating is assumed rather than demonstrated in neural architecture
**Implementational**	Describe the physical realization in the brain	Invoked in post hoc fashion (e.g., population codes, cortical hierarchies), but lacking falsifiable mappings or physiological specificity

## Post-Bayesian neuroscience: Dynamics, constraints, and interaction

The future of neuroscience lies not in decoding internal maps, but in understanding the terrain of interactions that shape them.

If the Bayesian brain hypothesis falters in its promise to mechanistically account for cognition, it is not for lack of mathematical elegance but for its failure to capture the situated, dynamical, and interaction-dominated nature of biological systems. Moving beyond this paradigm does not mean abandoning rigor or predictive power; it means shifting the center of gravity from inference to interaction, from static priors to evolving constraints, and from internal representations to systemic dynamics. The alternative is not chaos but a more grounded, empirically generative science of constraint-driven dynamics. To move beyond critique, **Table** 5 provides side-by-side examples of how constraint-based, dynamical approaches reframe core empirical domains traditionally modeled under Bayesian assumptions. These reorientations invite new modeling strategies, hypotheses, and empirical questions.

**Table 5:** How post-Bayesian frameworks rethink classic cognitive domains. This table contrasts the explanatory focus, assumptions, failure modes, and modeling strategies of Bayesian brain models versus interaction-dominant, constraint-based approaches. Examples are drawn from domains frequently used to support the Bayesian brain hypothesis

Domain	Bayesian explanation	Constraint-based alternative	Implications for modeling and methodology
**Multisensory integration**	The brain weights sensory cues based on prior reliability to form an optimal estimate	Cue weighting reflects bodily state, task demands, or environmental instability. Weighting adapts through time under metabolic or attentional constraints	Bayesian fits collapse variability into priors; dynamical models treat variability as a function of state-dependent coupling. Study trial-to-trial dynamics instead of average performance
**Perceptual illusions**	Deviations from veridical perception reflect strong priors overriding sensory input	Illusions emerge from ecological statistics or structural constraints of sensory surfaces. No inference is necessary	Bayesian accounts often fit priors post hoc; ecological/dynamical models study invariant detection and affordances. Test robustness across contexts
**Motor control and trajectory planning**	Optimal control under uncertainty: movement trajectories minimize expected cost	Movement emerges from real-time coordination of mechanical, energetic, and sensory constraints. Control is distributed, not centralized	Shift from inverse models to real-time feedback loops. Use coupled oscillators or tensegrity models instead of optimal feedback control
**Visual mismatch negativity (MMN)**	MMN reflects a neural prediction error in response to unexpected stimuli under a generative model	MMN arises from neural habituation, resonance, or phase resetting—not from hierarchical prediction	Instead of inferring priors, test for critical slowing, entrainment, or adaptation dynamics. Predict responses to repetition or variability, not “surprise.”
**Psychiatric symptoms (e.g., schizophrenia)**	Symptoms reflect misweighting of priors or prediction errors (e.g., hallucinations = over-weighted priors)	Symptoms result from destabilized inter-system coordination (e.g., disrupted brain-body-environment coupling, neuromodulatory breakdown)	Bayesian models are post hoc and descriptive; constraint-based models identify attractor breakdowns and loss of resilience. Use phase transition markers and whole-system indicators
**Emotion and interoception**	Affective states result from probabilistic inference over interoceptive signals. Valence = prediction error	Emotion emerges from dynamic regulation of constraints (e.g., thermoregulation, metabolic cost, proprioception). Affect = global coordination state	Rather than modeling internal inference, analyze physiological-environmental coupling. Use dynamical systems tools to track transitions between regulatory regimes

### From representations to real-time dynamics

Biological systems are not abstract inference engines. They are embodied, energy-consuming, open systems operating far from equilibrium. Their behavior emerges from richly structured, multi-timescale interactions among neural, physiological, and environmental processes. This shift—from inference over symbolic content to constraint-driven coordination—requires models that treat cognition not as the manipulation of probability distributions, but as the stabilization of activity within and across nested dynamical regimes.

In such a framework, the core unit of analysis is not the isolated variable or the internal model, but the evolving trajectory of the system within a space of possibilities shaped by constraints: mechanical, metabolic, informational, contextual. Constraints here are not merely boundary conditions or limitations; they are the generative architecture of behavior. Stability, flexibility, and adaptability emerge not from optimal inference, but from the real-time modulation of dynamics under shifting constraints.

### Modeling noise as signal: Complexity beyond Gaussian assumptions

Where Bayesian frameworks often rely on additive, stationary Gaussian noise for tractability, empirical observations consistently point toward noise that is multiplicative, state-dependent, heavy-tailed, and temporally correlated. These fluctuations are not nuisances to be averaged away; they are deeply intertwined with the system’s capacity for responsiveness and adaptation. Rather than modeling cognition as the minimization of prediction error within a narrow band of assumptions, post-Bayesian frameworks embrace noise as a source of exploration, variability, and emergent organization.

This perspective invites the use of simulation tools that do not impose a preordained structure on noise, but that allow interactions among nested constraints to shape complex behavior. State-space modeling, noise-driven dynamical systems, agent-environment coupling, and stochastic resonance models are promising pathways. Importantly, such tools do not seek to reproduce behavior through optimization, but to reproduce the *conditions* under which coherent, adaptive patterns emerge.

### Empirical commitments: from controlled inputs to naturalistic interaction

The research agenda for post-Bayesian neuroscience begins by shifting experimental emphasis. Rather than crafting ever more precise internal priors, we must develop methods to quantify how bodily states, environmental conditions, and temporal dependencies shape neural dynamics and behavioral outcomes. This includes:

- Measuring constraint modulation across timescales (e.g., metabolic fatigue, task-switching, proprioceptive reweighting)
- Analyzing coordination and entrainment across systems (e.g., brain-heart, posture-vision, movement-breath)
- Modeling behavior as the product of interacting dynamical systems with endogenous and exogenous inputs
- Designing tasks that probe adaptation under instability, uncertainty, or emergent constraint conflict

These approaches demand new methodological commitments:

- Longitudinal, high-dimensional recordings across neural and bodily systems
- Data analysis techniques sensitive to phase transitions, bifurcations, and nonstationarity
- Experimental designs that allow interaction, learning, and breakdown—not just well-constrained choice under fixed probabilities

### Toward a science of coordination, not computation

A post-Bayesian neuroscience must ask different questions. Instead of asking what internal model best explains observed behavior, we should ask what *interactive processes* give rise to coordination and breakdown. Instead of modeling perception as posterior estimation, we should investigate how perceptual systems stabilize amidst competing constraints. Instead of using priors to constrain likelihoods, we should examine how history, development, fatigue, and context modulate the attractor landscape of behavior.

This is a science of constraints and co-regulation, not representation and error correction. It privileges fluidity over optimality, adaptability over accuracy, and coordination over computation. And crucially, it returns neuroscience to the task of understanding real organisms acting in real environments—not in the language of ideal observers, but in the dynamics of lived interaction.

This shift will not be easy. It requires new tools, new metaphors, and new standards for theoretical rigor. But it promises a neuroscience that is empirically anchored, biologically plausible, and better equipped to confront the complexity of embodied cognition. A neuroscience not of Bayesian brains, but of living, learning, and dynamically constrained bodies in motion. Where Marr’s computational level invites abstract formulations, the algorithmic and implementational levels demand specificity. Post-Bayesian models must engage these levels directly, grounding dynamics in biologically plausible mechanisms rather than idealized probabilistic reasoning.

## Beyond Bayesianism

A science that fears contradiction is not a science that seeks truth but one that preserves dogma. The path forward requires not more flexible Bayesian models, but the courage to abandon mathematical convenience for biological reality.

This critical examination of the Bayesian brain hypothesis is not intended to dismiss its contributions or deny its insights. Rather, it aims to clarify its proper status and limitations in the scientific study of mind and brain. To conclude, I offer specific recommendations on how cognitive neuroscience might move forward in light of these critiques.

**Fig. 3:**
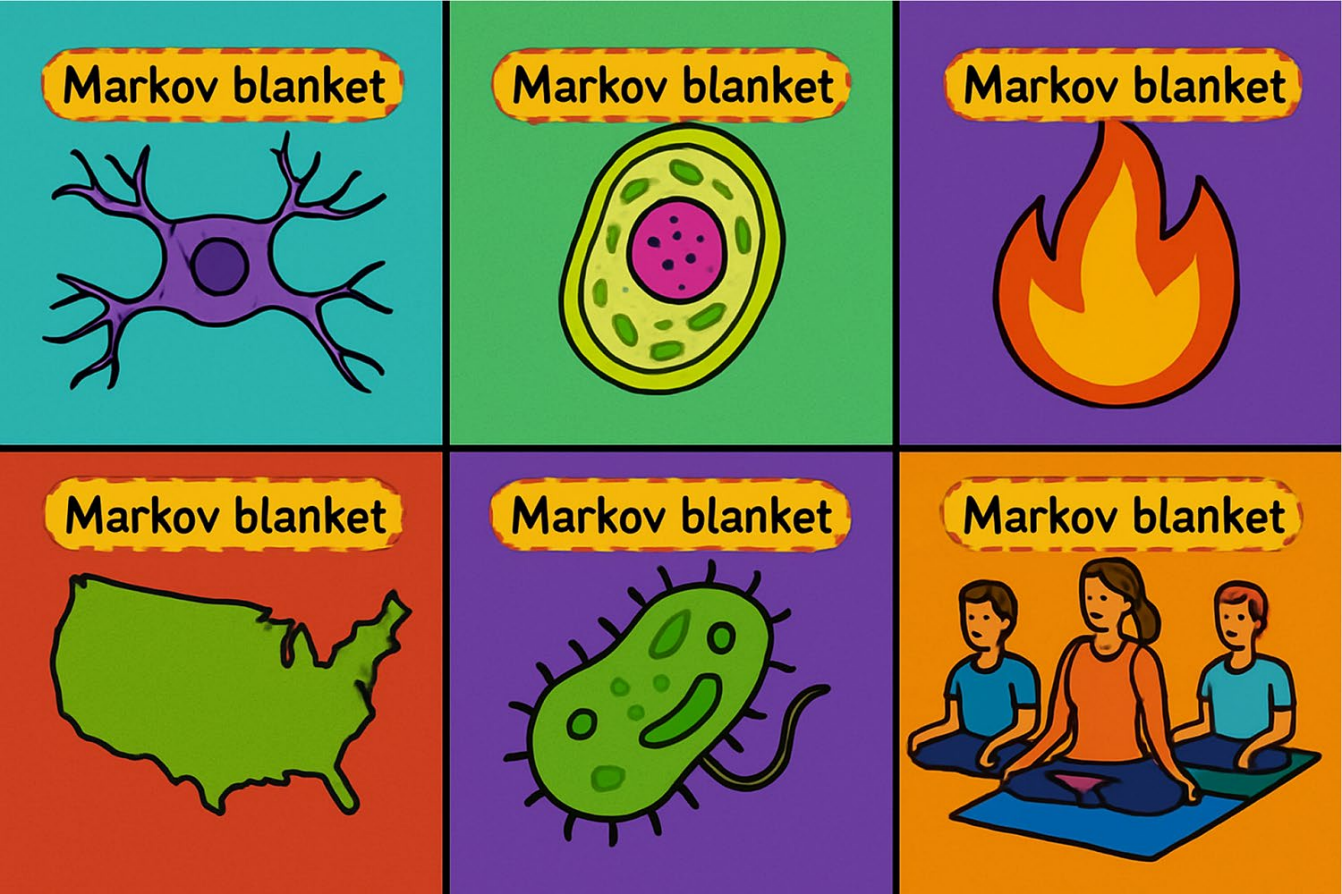
Illustrative examples of the conceptual overextension of the “Markov blanket” construct across ontologically disparate systems. While originally formalized as a boundary condition in graphical models of statistical dependency, the term has increasingly been applied to biological, cognitive, and even sociocultural entities—from neurons and cells to flames, microbes, nation-states, and human collectives. This proliferation raises concerns about the dilution of mechanistic specificity and the erosion of explanatory constraint within theoretical neuroscience

First, we must recognize that the Bayesian brain, while influential and mathematically sophisticated, risks becoming pseudoscientific when it sheds the two most important scientific requirements: falsifiability and mechanistic grounding. Proponents must specify concrete neural implementations and falsifiable predictions to maintain scientific integrity. For example, falsification would require identifying conditions under which a system’s behavior consistently violates Bayesian predictions—such as when neural populations fail to signal prediction error in response to surprising stimuli, or when behavior ignores prior probabilities in controlled decision tasks where such information is available and relevant. While some predictive coding and active inference models have been used to simulate observed neural responses, such simulations typically rely on adjusted priors and free parameters post hoc, rather than offering risky, *a priori* predictions that could be decisively falsified. For example, rather than merely claiming that perception follows Bayesian principles, researchers should identify specific neuronal populations that encode probability distributions and specify how these distributions are updated through synaptic mechanisms. Karl Friston’s Free Energy Principle, in particular, needs clearer operational definitions and greater specificity about what would constitute disconfirming evidence. Although terms like “Markov blanket” (**Fig.** 3) and “generative model” are often presented as mechanistic constructs, they frequently function as abstract formalisms without directly mapping specific neurophysiological structures or processes.

Second, we should embrace theoretical pluralism not as a weak compromise but as a principled alternative to the hegemony of Bayesian approaches. Visual neuroscience provides a case in point: while predictive coding models have gained prominence, robust empirical research continues to emerge from alternative frameworks—such as resonance theories, neural synchrony models, and dynamic field approaches—that make no reference to Bayesian inference. These models often emphasize dynamical coordination, oscillatory coupling, or stability through feedback rather than probabilistic computation. Embodied approaches like those of Thelen and Smith, the ecological psychology of Gibson, and dynamical systems models developed by Kelso offer explanatory frameworks that directly challenge, rather than complement, Bayesian formulations. These approaches proceed from fundamentally different premises about the nature of cognition—emphasizing direct perception, self-organization, and emergent dynamics rather than internal models and inference—and have generated productive research programs without the conceptual baggage of Bayesian metaphysics.

Third, it is not sufficient to defend the Bayesian brain hypothesis by appealing to a misunderstood scope. This paper does not critique a caricature, but rather the dominant rhetorical and modeling practices associated with Bayesian brain theories. If the theory continually shifts between metaphor and mechanism, between universal law and specific model, then its boundaries must be clearly defined before its scientific status can be responsibly assessed. Future theories of mind and brain should prioritize biological plausibility, empirical testability, and theoretical humility. Sampling-based approximations proposed by researchers like Fiser, Berkes, and Orbán represent promising steps toward biologically realistic implementations of probabilistic inference. Yet even in approximation mode, these models often leave unclear what exactly is being approximated, how such approximations are implemented neurally, and under what conditions the brain diverges from or conforms to Bayesian logic. Proponents often respond that approximate inference mitigates concerns about biological plausibility. However, invoking approximation does not clarify the underlying process—unless the approximations themselves are mechanistically specified and empirically constrained, the theory risks being shielded from disconfirmation. This ambiguity allows Bayesian claims to persist in the absence of direct empirical support. Without such specificity, the models remain computationally elegant but biologically opaque. Greater scrutiny is especially warranted for frameworks like active inference: while Adams, Shipp, and Friston have proposed that neuromodulators encode precision, these claims remain largely post hoc and have not yielded novel, specific, and empirically validated predictions. This exemplifies the core problem critiqued throughout this paper—the tendency to retrofit existing findings into Bayesian language without offering falsifiable hypotheses. Going forward, cognitive neuroscience must prioritize approaches that genuinely connect abstract computational principles to concrete neural mechanisms and produce truly testable experimental predictions—something the Bayesian brain literature has largely failed to deliver.

Finally, we should embrace the messiness and complexity of embodied, context-sensitive cognition without forcing it into ill-fitting probabilistic frameworks. While Cisek and Kalaska’s work on affordance competition offers valuable insights into how the brain represents action possibilities, attempts to subsume such approaches under predictive processing frameworks (as in Clark’s work) or to reframe emotion in terms of Bayesian inference (as in Barrett’s constructed emotion theory) often exemplify precisely the problem this paper critiques: retrofitting non-Bayesian insights into Bayesian language without adding explanatory value. The brain has evolved not to implement Bayes’ theorem but to coordinate adaptive behavior in complex, uncertain environments, typically through simpler, more robust mechanisms. The heuristic strategies documented by Gigerenzer and colleagues demonstrate how effective decision-making can emerge without the computational burden of full probabilistic processing. It challenges the core premises of the Bayesian brain hypothesis rather than complementing it.

The path forward requires moving decisively beyond the Bayesian paradigm rather than continuing to grant it special status. Clark’s predictive processing framework, despite its claims of integrating action and embodiment, ultimately subordinates these elements to probabilistic inference, maintaining the very problems of biological implausibility and unfalsifiability that this paper critiques. Similarly, Tenenbaum’s probabilistic graphical models in cognitive development research exemplify the post-hoc modeling approach that fits parameters to existing data without generating truly novel predictions—merely redescribing phenomena in Bayesian terminology without advancing mechanistic understanding. This shift demands not just methodological adjustment, but a fundamental reorientation of explanatory priorities—from abstract, probabilistic constructs toward models grounded in the material, dynamical, and context-sensitive nature of real brains in real bodies. Rather than attempting to salvage Bayesian approaches, cognitive neuroscience would benefit from fresh theoretical frameworks that start from biological realities and embodied cognition, rather than from mathematical formalisms that must be awkwardly retrofitted to neural systems.

Cognitive neuroscience can develop more nuanced, integrative, and pluralistic approaches to understanding the mind–brain relationship by acknowledging the fundamental limitations of the Bayesian approach and actively embracing a wider range of theoretical alternatives. As Marr emphasized in his influential levels-of-analysis framework, a central and enduring challenge is to bridge computational theory, algorithmic description, and biological implementation in a mechanistically specific and empirically testable way. Meeting this challenge requires navigating carefully between metaphor and mechanism, as well as between mathematical elegance and biological plausibility. Scientific progress depends on building theories that are both explanatory and empirically grounded. In the end, the Bayesian brain hypothesis may have served as a useful metaphor—but the future of neuroscience lies in the dynamic, constraint-bound interplay of bodies, brains, and worlds.

### Final recommendations: A summary in five points

To close, we distill the main critiques and suggestions of this paper into five concise, actionable points for theoretical neuroscience:

1. **Demand falsifiability and mechanistic grounding.** Theories of brain function must make risky predictions and specify biologically plausible mechanisms. Abstract elegance is not enough.
2. **Embrace theoretical pluralism.** Competing frameworks—such as dynamic systems theory, ecological psychology, and embodied cognition—offer rich, empirically grounded alternatives to the Bayesian brain hypothesis.
3. **Clarify scope and level of explanation.** The Bayesian brain often blurs metaphor and mechanism, oscillating between universal principles and specific implementations. Clear theoretical boundaries are essential.
4. **Avoid post hoc rationalization.** Approximate inference, flexible priors, and retrospective model fitting can immunize the theory from refutation. Scientific progress requires vulnerability to being wrong. This critique applies most directly to generalized or unfalsifiable uses of Bayesian modeling, rather than to all Bayesian models.
5. **Center biology, not formalism.** Theories must begin with the physical and dynamical constraints of real neural systems—not with formalisms that require biology to conform after the fact.

These recommendations aim to realign cognitive psychology and neuroscience with the principles of empirical accountability, biological plausibility, and conceptual clarity. By following these recommendations, cognitive neuroscience can develop more rigorous, biologically grounded, and empirically accountable theories of brain function. The future of our field depends not on the elegant mathematics of inference machines but on understanding the messy reality of embodied cognition.

## The final blow: A crucial experiment and the limits of the Bayesian metaphor

A framework that accounts for everything, regardless of outcome, is less a scientific theory than a metaphysical comfort blanket.

### Concrete neural predictions, not post hoc fits

To deliver the definitive challenge to the Bayesian brain hypothesis, we propose a single, tightly controlled empirical test that targets the hypothesis in its strongest, most mechanistic form. This “crucial experiment” aims to show whether the brain really implements Bayesian-style computations—or whether such claims persist only by post hoc reinterpretation and parameter-tweaking. If the Bayesian brain hypothesis is to remain viable as a mechanistic explanation, it must pass a decisive empirical test—one that makes clear, pre-registered predictions about neural dynamics under well-defined conditions.

**Setup:** Identify a particular cortical or subcortical circuit that Bayesian/predictive-coding accounts explicitly claim encodes priors, likelihoods, or precision weights during a well-studied perceptual task. The key is to specify, *in advance*:

1. Which neuronal population (*layer, area, microcircuit*) encodes the “prior belief,”
2. Which population encodes “prediction errors,”
3. How “precision” modulates firing rates or synaptic gains,
4. Precisely how these populations will respond if they implement Bayes’ rule under varying prior probabilities and likelihood manipulations.

**Crucial test:** Employ real-time neural recordings (e.g., multi-electrode arrays, two-photon calcium imaging) while systematically manipulating both the prior probability and the reliability (*likelihood*) of the sensory cue. For instance, a strong prior via a predictive cue can be introduced, and then the actual likelihood or degraded sensory signal can be abruptly altered.

In practical terms, researchers could train animals in a visual-cue task with explicit priors (e.g., 80% of trials have a bright stimulus, 20% a dim one). We would measure multi-electrode activity in cortical region X, hypothesized to represent the prior. We would also record from cortical region Y, hypothesized to encode precision-weighted prediction errors, using real-time decoding. The Bayesian model must specify, *a priori*, how spike rates and local field potentials in regions X and Y should systematically shift in response to manipulations of likelihood. If we fail to see the predicted parametric shift in neural activity in real time—and cannot salvage it with post hoc parameter tweaks—this would falsify the strong Bayesian claim.

- **If the Bayesian brain is literally correct**: We should observe immediate, lawful shifts in the *prior-encoding* population that precisely match the updated posterior distribution, with no new free parameters introduced post hoc.
- The “prediction error” populations *must* show systematic, monotonic changes that reflect the mismatch between the experimenter-manipulated priors and the newly introduced likelihoods.
- These changes in firing or synaptic gain must match the timescale and hierarchy predicted *a priori* by the Bayesian model.

Any major divergence between the *pre-registered* predictions and actual neural data would be fatal to the hypothesis, unless proponents resort to the usual escapes (e.g., “the priors were really something else”). But such reparameterization would only confirm that the theory remains “safe” by becoming *unfalsifiable*. Unless proponents can answer the empirical and mechanistic challenges summarized in **Box 2**, the Bayesian brain should be reclassified not as a scientific theory, but as a durable metaphor—valuable in pedagogy, but inert in biology.

**Table:** 

**Box 2: Questions the Bayesian brain hypothesis must answer—but can’t**	
1. **Where in the brain are priors stored, and how are they represented at the circuit level?**	
The framework often refers to priors, but never specifies where or how they exist biologically.	
2.**Which neural mechanisms encode the full probability distributions required for Bayesian updating?**	
Models assume distributions, but neurons fire spikes—not functions over state spaces.	
3.**How does the brain compute likelihood functions in real-time under metabolic constraints?**	
There’s no evidence of likelihood computation in real circuits under ecological conditions.	
4.**What, exactly, is the generative model in a given task, and how is it implemented?**	
“Generative model” is often invoked as a black box—never mapped onto structure or process.	
5.**How do we distinguish neural evidence of prediction error from adaptation or surprise?**	
EEG/fMRI correlates are too coarse to adjudicate between Bayesian and non-Bayesian accounts.	
6.**When does the Bayesian brain make a wrong prediction—and how is that measured biologically?**	
Without a theory of “Bayesian failure,” the framework is immune to error and thus unscientific.	
7.**How would we know if the brain is** *not* **Bayesian?**	
If no empirical result could falsify the framework, then the framework is no longer science.	

### Escaping the “Heads I Win, Tails You Lose” trap

A persistent problem is that whenever data fail to match Bayesian predictions, proponents retreat to:

1. New “approximation” claims,
2. Suboptimal inference or “algorithmic constraints,”
3. Ad hoc changes to priors,
4. Shifting the explanation to some other hierarchical level.

A truly decisive experiment would forbid these infinite get-out-of-jail-free cards by demanding:

- A *single* form of approximate inference specified in advance,
- A *fixed* prior for each condition,
- *No* indefinite hierarchical recourse.

Only if the pre-registered predictions align with the measured neural data can we claim real Bayesian updating in the literal sense.

### When a theory becomes myth

A theory that deflects every mismatch by revising priors or recasting “precision parameters” is no longer empirical science but a *metaphor*—or at worst, a scientific *myth*. The final blow to the Bayesian brain emerges precisely when the hypothesis shows it can “explain away” *any* neural or behavioral result by retroactive tuning. Such boundless flexibility ensures it “survives” but only by sacrificing actual predictive power—a framework that cannot be wrong also cannot tell us anything new about how the brain really works.

### Accepting the consequences

If the Bayesian brain hypothesis is genuinely falsifiable, then its proponents should eagerly welcome and design a crucial, pre-registered experiment that explicitly ties each core theoretical quantity—such as priors, likelihoods, prediction errors, and precision weights—to well-defined, identifiable neural populations and circuits in specific, testable ways. Should that experiment fail to confirm the predicted computations, the strong Bayesian claim collapses. Declining to propose or run such a decisive test, or retreating to indefinite “as-if” language, would itself be the final, tacit concession: the Bayesian brain is not an implementational model of cognition but a rhetorical flourish—valuable as metaphor, but scientifically inert as a mechanistic account—an idea immune to failure, and thus unfit for science.

## Epilogue: The map and the terrain

The brain is not a statistician calculating posterior probabilities in a vacuum. It is a body in the world, learning to move well enough to stay alive—a project for which Bayes’ theorem is neither necessary nor sufficient.

The Bayesian brain may have offered a seductive map—clean, coherent, and mathematically elegant. That elegance, however, often conceals the unruly, dynamic complexity it claims to summarize. But as every cartographer knows, maps are not the territory. The brain’s terrain is rugged, adaptive, and radically embodied. It pulses with histories, constraints, and interactions that no prior or posterior can quite capture. To understand minds, we must walk the ground—muddy, nonlinear, and alive.

## Data Availability

Data sharing does not apply to this article as no new data were created or analyzed in this study.

## Appendix: Historical glossary of Bayesian expansion

This appendix traces the conceptual drift of the Bayesian brain hypothesis from its origins in sensory processing to its current status as a purportedly universal principle. The timeline reveals not just historical development but rhetorical expansion—a pattern of widening scope accompanied by weakening constraints. Each new domain added to the Bayesian framework brings mathematical elegance but raises empirical concerns about overextension and unfalsifiability.

This appendix offers a chronological overview of how the Bayesian brain hypothesis has expanded across domains (**Table** 6). What follows is not merely a timeline, but a rhetorical map of a framework growing too fast to be critically metabolized. The timeline may serve as both a historical reference and a cautionary tale.

**Table 6:** History of Bayesian expansion. This timeline highlights how the Bayesian brain hypothesis has drifted across domains over the past two decades. Each entry marks a turning point in the scope, ambition, or rhetorical framing of Bayesian models, illustrating how an initially targeted theory of sensory processing became a universal template

Period	Domain	Key works	Conceptual shift
1999–2003	Early vision	(Ernst and Banks 2002; Lee and Mumford 2003; Rao and Ballard 1999)	Perception as hierarchical prediction. Initial formulation of predictive coding
2004–2007	Sensorimotor integration	(Knill and Pouget 2004; Friston 2005)	Multisensory cue integration framed as Bayesian weighting. Optimally fused estimates
2007–2010	General cognition	(Friston 2010; Doya 2007)	Free Energy Principle introduced. “Bayesian brain” extended to decision, emotion, action
2010–2015	Psychiatry & emotion	(Adams et al. 2013; Hohwy 2013)	Predictive processing applied to psychosis, anxiety, autism. Uncertainty reframed as affect
2015–2018	Artificial intelligence	(Friston et al. 2016; Buckley et al. 2017)	Active inference linked to AI/robotics. Bayesian control and generative models fused
2018–2023	Evolution & cell biology	(Hipólito et al. 2021; Ramstead et al. 2023)	Markov blankets reinterpreted as boundaries of all living systems. Bayesian selfhood
2023–Present	Everything	(Numerous)	Bayesian framing applied to candle flames, phototaxis, immune cells, and cosmology. Universality normalized

As the scope of Bayesian rhetoric has widened, its empirical constraints have thinned. This drift raises urgent questions about whether theoretical breadth has outpaced scientific utility. The result is a theory that increasingly functions as a universal metaphor rather than a testable scientific model.
